# Modeling the Seasonal Adaptation of Circadian Clocks by Changes in the Network Structure of the Suprachiasmatic Nucleus

**DOI:** 10.1371/journal.pcbi.1002697

**Published:** 2012-09-20

**Authors:** Christian Bodenstein, Marko Gosak, Stefan Schuster, Marko Marhl, Matjaž Perc

**Affiliations:** 1 Department of Bioinformatics, Friedrich Schiller University Jena, Jena, Germany; 2 Faculty of Natural Sciences and Mathematics, University of Maribor, Maribor, Slovenia; École Normale Supérieure, College de France, CNRS, France

## Abstract

The dynamics of circadian rhythms needs to be adapted to day length changes between summer and winter. It has been observed experimentally, however, that the dynamics of individual neurons of the suprachiasmatic nucleus (SCN) does not change as the seasons change. Rather, the seasonal adaptation of the circadian clock is hypothesized to be a consequence of changes in the intercellular dynamics, which leads to a phase distribution of electrical activity of SCN neurons that is narrower in winter and broader during summer. Yet to understand this complex intercellular dynamics, a more thorough understanding of the impact of the network structure formed by the SCN neurons is needed. To that effect, we propose a mathematical model for the dynamics of the SCN neuronal architecture in which the structure of the network plays a pivotal role. Using our model we show that the fraction of long-range cell-to-cell connections and the seasonal changes in the daily rhythms may be tightly related. In particular, simulations of the proposed mathematical model indicate that the fraction of long-range connections between the cells adjusts the phase distribution and consequently the length of the behavioral activity as follows: dense long-range connections during winter lead to a narrow activity phase, while rare long-range connections during summer lead to a broad activity phase. Our model is also able to account for the experimental observations indicating a larger light-induced phase-shift of the circadian clock during winter, which we show to be a consequence of higher synchronization between neurons. Our model thus provides evidence that the variations in the seasonal dynamics of circadian clocks can in part also be understood and regulated by the plasticity of the SCN network structure.

## Author Summary

Circadian clocks drive the temporal coordination of internal biological processes, which in turn determine daily rhythms in physiology and behavior in the most diverse organisms. In mammals, the 24-hour timing clock resides in the suprachiasmatic nucleus (SCN) of the hypothalamus. The SCN is a network of interconnected neurons that serves as a robust self-sustained circadian pacemaker. The electrical activity of these neurons and their synchronization with the 24-hour cycle is established via the environmental day and night cycles. Apart from daily luminance changes, mammals are exposed to seasonal day length changes as well. Remarkably, it has been shown experimentally that the seasonal adaptations to different photoperiods are related to the modifications of the neuronal activity of the SCN due to the plasticity of the network. In our paper, by developing a mathematical model of the SCN architecture, we explore in depth the role of the structure of this important neuronal network. We show that the redistribution of the neuronal activity during winter and summer can in part be explained by structural changes of the network. Interestingly, the alterations of the electrical activity patterns can be related with small-world properties of our proposed SCN network.

## Introduction

The circadian rhythm is a 24 h rhythm which can be found in many organisms ranging from cyanobacteria and fungi to mammals [1], [2], [3]. There is a huge interest in studying the circadian rhythm because of the well-known effects of jet-lag after traveling and diseases related to shift-work [4], [5]. In mammals the major pacemaker is the suprachiasmatic nucleus (SCN), which synchronizes all peripheral clocks in the body and controls the overall behavior [6], [7], [8]. It is a small region in the hypothalamus located below the third ventricle and directly above the optic chiasm. Light is the major entrainment factor of the SCN. An important dynamical property of the SCN is that it adapts to different photoperiods in summer and in winter [9], [10]. This means that the behavioral activity should be longer in summer days than in winter days, which is advantageous for the organism [11], [12].

The SCN is a symmetric structure, consisting of approximately 20 000 neurons, where each part is usually classified into a ventrolateral (VL) and dorsomedial (DM) region [13], [14], [15]. Cells in the VL region mainly express the neuropeptide, vasoactive intestinal peptide (VIP), comprising around 24% of the SCN neurons in rats [15], and receive the light information from photosensitive retinal ganglion cells [7], [16]. However, also other cell types have been identified that show light induced gene expression. For example in hamsters, cells expressing calbindin (CalB) are responsible for the mediation of light information [17]. Importantly, it has been found that these cells are nonrhythmic and uncoupled from each other [17], 18,19,20. In contrast to this cells in the DM region, expressing mainly vasopressin (AVP) are rhythmic but do not receive light input [17]. It has been shown that separating a part of the DM region leads to nonsynchronous rhythms in the individual cells [21], indicating that the coupling in the DM region is not sufficient to synchronize these cells. Indeed it has been shown that interactions between cells in this region are restricted to short-range connections [22]. On the other hand long range connections of neurons in the VL region to neurons in the DM region have been identified [13], [23], which seem to be important to synchronize cells in the DM and VL region to each other and ensure entrainment of DM cells to an external light-cycle. We are aware that the distinction into a VL and DM region is an oversimplification and not that clear in other species than rats since the distribution of neurotransmitters and retinal inputs shows a more complex spatial organization [17], [24], [25]. Nevertheless, since our study is a first attempt to model the influence of long-range couplings we aim at a simple and manageable model. Moreover, the separation into a “core" part, represented by the VL cells, and a “shell" part, represented by DM cells, is commonly accepted also for other rodents [17].

The importance of the neuronal network circuitry mediated by chemical synaptic interactions as opposed to simple global coupling has been demonstrated in several experimental studies [13], [21]. All these studies show that the functioning of the SCN and the regulation of the circadian rhythm in general is based on a very complex neuronal network consisting of short- and long-range connections. The question arises how the structural properties of this complex neuronal network are linked with the electrical activity of the SCN, which is known to crucially affect the circadian gene expression [26].

It is known that the seasonal adaption of the SCN is closely related to its electrical activity [9], [10]. In particular, single cells in the SCN have a peak in electrical activity (measured as firing activity) from 4–5 h, regardless of monitoring in winter or summer conditions [9], [27], [28], [29], [30], [31]. Therefore, it has been suggested that the phase distribution of electrical activity inside the SCN neurons, which is narrower in winter than in summer, leads to a shortened behavioral activity in winter [10]. Different phase distributions in winter and summer have been previously modeled by introducing delay times in the synaptic connections [32].

Here we hypothesize that the adaptation to seasonal changes in the phase distributions can also be related to changes in the structural properties, i.e., the topology and coupling of the complex neuronal network of the SCN. This is a reasonable hypothesis since, as already mentioned in the previous paragraph, the individual neurons do not change their electrical activity in summer and in winter. Many studies have already examined the network topology of the SCN in various manners [32], [33], [34], [35], . These studies use different single cell models that range from a generic van-der-Pol oscillator [32], [34] to more detailed biochemical models [36], [37], [38]. Moreover, they model coupling between the cells in different ways. Whereas some studies considered homogenous coupling between all cells [33], [35] others take into account the heterogeneity in the SCN cellular network [34], [36], [37], [38]. Furthermore, Vasalou et al. [37] have reported that a small-world network architecture of the SCN can firmly mimic the dynamical behavior of mean-field coupled models, but is on the other hand much more efficient in terms of connectivity cost. Also Hafner et al. [38] in a recent study analyze different network topologies with respect to rhythm output and jet-lag adaptation and find that coupling different network topologies leads to robustness of the overall rhythm with respect to perturbations. Nevertheless, all of the abovementioned studies were mainly focused on the synchronization and amplitude properties, entrainment and robustness of the SCN.

In our study, we focus on the role of long-range connections between the neurons, as they are known to lead to networks characterized by small-world properties [39], [40]. Moreover, in jet-lag experiments evidence was found for a connection between the ventral and dorsal part of the SCN [10], indicating a role for long-range connections between both parts. Using our model, defined by coupled ordinary differential equations, we show that the number of long-range connections between the cells in the VL and DM region is a fine-tunable parameter to adjust the phase distribution and consequently the length of behavioral activity. Our results thus indicate that the seasonal summer/winter dynamics of the circadian clocks can effectively be regulated by the plasticity of the SCN network structure.

## Materials and Methods

### Single cell oscillator

It has been shown in mathematical models that the electrical activity measured as the firing rate of neurons is directly related by a threshold mechanism to the underlying molecular clockwork composed of transcriptional and translational feedback loops [41], [42]. The firing rate is then encoded into the release of neurotransmitter via synapses that in turn affect the underlying molecular clockwork by a cascade involving Ca^2+^, cAMP and CRE elements in the promotor region of *Per* and *Cry* [13]. Here, we are using a generic amplitude-phase oscillator model that was used in recent studies on the entrainment and the importance of coupling in circadian rhythms and is commonly referred to as the Poincaré oscillator [43], [44], [45]. The advantage of this model is its few independent parameters namely the radial relaxation rate 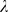 and the relative amplitude *A*. It has been shown that many high-dimensional oscillator models can be reduced to simple two-dimensional amplitude-phase oscillator models [46]. The parameters for this model have been taken from model fits to measurements from single dissociated SCN cells showing a log-normal distribution, with an average value of 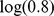/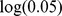 and standard deviation 0.5/0.4 of the underlying normal distribution for *A* and 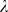, respectively [47]. Non-rhythmic cells were modeled with the amplitude set to zero *A* = 0, resulting in a damped oscillator [47]. To mimic the spike-like electrical activity of 4–5 h, we adjusted the model as suggested in [43], with the parameter 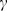 controlling the phase velocity change set to 2. The oscillator in the amplitude-phase (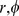) representation is given by:
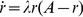
(1)

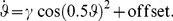
(2)Here the offset parameter is chosen in such a way that the period of the individual oscillators is Gaussian distributed around 24 h with a standard deviation of 3 h. This ensures that most of the intrinsic periods are in a range of 18–30 h, as experimentally observed [48].

### Oscillator network

**Figure 1:**
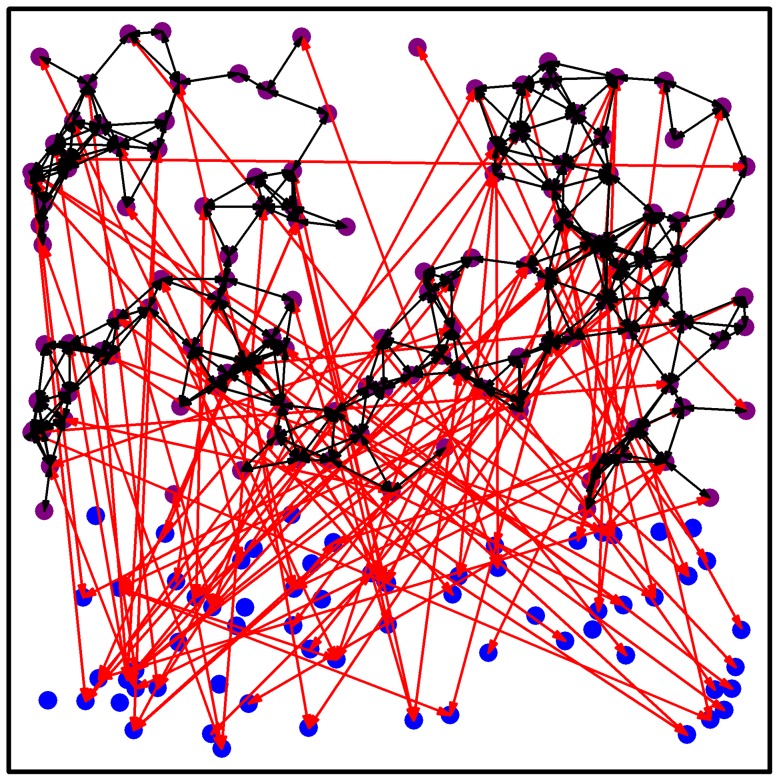
Characteristic network architecture of the SNC. The number of neurons is 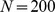 and 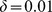. Blue circles denote neurons in the ventrolateral (VL) while violet circles denote neurons in the dorsomedial (DM) region. Black arrows depict short-range connections and red arrows depict long-range connections.

To reduce computational costs our main SCN model consists of 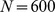 cells. However, to check whether our results are independent of the system size, we later also consider a network of 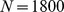 cells. To reflect the light-receiving and non-rhythmic cells (*A* = 0) in the VL region we distributed 1/3 of the neurons in the lower region randomly without any connections between them. The other 2/3 of the rhythmic cells was distributed randomly above. These cells were connected as a random geometric graph [49], [50]. In particular, for simplicity an identical radius range 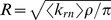 for all cells was chosen, where 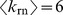 signifies the average degree of the random geometric network and 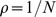 is the density of the cells in the region. If two cells fall within each other's range then they are connected. To analyze the importance of the long-range connections between the VL and the DM region they were added with an adjustable probability 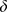, similar as in a previous study on small network properties in the SCN [37]. Moreover, since we cannot exclude long-range connections between cells within the same region (DM, VL), these were also added but with a ten times smaller probability (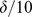). For simplicity we only considered bidirectional coupling in all cell-to-cell connections. A characteristic network structure is shown in Figure 1. We use a linear, local mean field coupling model that averages the inputs into each cell and adds these to the oscillator in Cartesian coordinates. It has been used in previous SCN coupling studies [36], [37]. The terms:
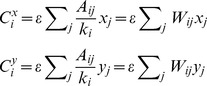
(3)are added to Eqs. 1–2 after transformation into Cartesian coordinates. Here 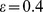 defines the coupling strength, 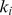 is the degree of node *i* and 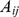 is the *ij*-th element of the adjacency matrix, whose value is 1 if the oscillators are coupled, whilst otherwise the value is 0. The oscillators are not coupled to themselves (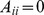).

### Light input

The light input into our model was simulated by adding the light signal to the *x*-coordinate of each oscillator cell in the VL region. The light signal was modeled as a square shaped pulse with a period of 24 h and adjustable width 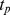, which enables us to simulate different photoperiod lengths. In Section 6 in Text S1 we analyzed the entrainment capacities of a single model oscillator and found that only forcing in the *x*-coordinate is suitable for entrainment, since forcing in *y* leads to a shift of the intrinsic period to lower periods (see Eq. 44 in Text S1). In all calculations we used a fixed amplitude 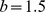 for the periodic light input.

### Phase response curves

Phase response curves (PRCs) are a very useful tool to characterize circadian rhythms [51]. They measure the advance or delay of the clocks phase to a perturbation applied at different times of the day. The PRC is measured in our model by applying a light-pulse of 4 h duration and amplitude 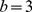 to the free-running rhythm. The PRC is then scaled to circadian time (CT0 to CT24) by entraining the organism to an external rhythm and taking the maximum of the 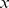 variable as a phase reference point [51]. The infinitesimal or instantaneous PRCs (iPRCs) describe the phase response to an infinitesimally short and small light pulse [52] of each individual oscillator and can be calculated from adjoint equations [53], [54], [55], [56] (cf. Section 1 in Text S1). Importantly, they allow disassembling the overall PRC, which can be used to deduce factors affecting its magnitude or shape.

### Network measures

Due to the long-range connections interaction networks with small world properties can emerge [37], [40]. Their existence is characterized by a relatively high efficiency *E*, which serves as an indicator of the traffic capacity of the network and is defined as follows:
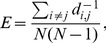
(4)where 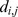 is the length of the shortest path from unit *i* to unit *j*. It should be noted that *E* is inversely related to the average shortest path length, but is numerically easier to use for the estimation of topological distances between elements of disconnected graphs. On the other hand, the cliquishness of a typical neighborhood in small-world networks is large. This characteristic is usually quantified using the clustering coefficient *C*, which is defined as follows: If the node degree (the number of neighbors) of a vertex *i* is denoted by 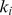, there are 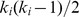 possible links between these neighbors. One commonly denotes 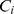 as the fraction of those links that are present in the graph and *C* is defined as the average of 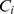 over all the vertices. Furthermore, it has been shown [57], [58] that the product 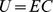 is a suitable indicator for the optimal small-world network structure, because 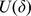 has its maximum in the region of 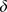, where a proper ratio between the clustering and the efficiency is achieved. It should also be noted that it makes sense to argue about small-world characteristics only for low enough values of 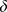 (i.e. 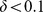) [58]. Above this value the interconnectivity becomes too large and the coupling behaves more as a mean-field type.

### Synchronization and spectral graph analysis

The synchronization behavior of coupled oscillators for small coupling strengths can be deduced from the eigenvalues of the Laplacian matrix of the network [45], [59], [60] (see Section 5 in Text S1 for further explanation). In Sections 2, 3 and 4 in Text S1 we establish a theory for the phase synchronization of weakly coupled heterogeneous oscillators in an arbitrary network by using the phase-reduction method introduced by Kuramoto [61]. We assume that the heterogeneity in the oscillators and their coupling is small and Gaussian distributed. This is a simplification of our considered network structure because it contains a mixture of damped and self-sustained oscillators and thus a rather large heterogeneity between these two groups. Nevertheless, the analytic results lead to deeper insights into the system and can still be helpful to understand the systems dynamics. For the most general case we find that if the in-phase locking of oscillators is stable, the variance of the stable phase distribution is mainly determined by near-zero singular values of the coupling matrix ***M***, which determines the dynamics of deviations from the synchronized state. If the coupling between the oscillators is additive and similar, the coupling matrix is identical to the Laplacian matrix ***L*** of the network up to multiplication by a constant factor. The Laplacian matrix is defined as:
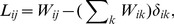
(5)where 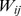 is the weighted adjacency matrix from Eq. (3) and 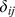 is the Dirac delta function. Furthermore, if the Laplacian ***L*** is symmetric due to a bidirectional coupling of oscillators the singular values can be replaced by the eigenvalues of ***L***. However, in our case the matrix ***L*** in Eq. (5) is not symmetric because of the local mean field coupling. Thus, we calculated the singular values after removing all completely disconnected oscillators from the network because each disconnected oscillator leads to a trivial zero singular/eigenvalue in the spectrum.

### The Goodwin model

In order to generalize our findings we additionally verify how the SCN network behaves when a more complex model for circadian oscillations that, to a certain extent, takes into account molecular aspects of the circadian clock describes the dynamics of the individual cells. The mathematical formalism used to describe the dynamics of individual oscillators is based on the theoretical framework of Goodwin [62] and its extended version proposed by Gonze et al. [35]. The dynamics of the *i*-th cell is governed by the following set of differential equations:
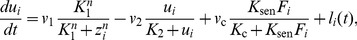
(6)

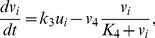
(7)

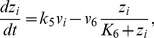
(8)

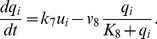
(9)In Eqs. (6–9) 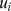 denotes the clock gene mRNA which produces a clock protein 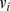 which, in turn, activates a transcriptional inhibitor 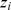. Moreover, 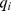 signifies the neuropeptide serving as a means for intercellular communication. In particular, the neurotransmitter level 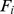 affects the clock gene transcription (see Eq. (6)), whereby the neurotransmitter interactions are determined by the network structure:
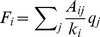
(10)The term 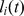 in Eq. (6) represents the light signal, which is applied only to cells in the VL region and is modeled as a square shaped pulse with a period of 24 h and adjustable width 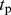 and amplitude 0.01. To mimic the nonrhythmic behavior of cells in the VL region we set 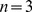 for 1/3 of the neurons, whereas in the upper DM region the Hill coefficient for all the cells is set to 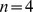, so that they exhibit self-sustained oscillations. However, a reduced Hill coefficient results in a decrease of the inherent oscillator frequency. For that reason we adjusted the values for the degradation rates of the clock gene, in order to achieve an inherent period around 24 h for both self-sustained and damped oscillators. In particular, in the VL region we set 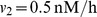, whereas in the DM region we chose 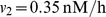. Other parameters used in our calculations, except for the coupling strength 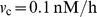, were chosen according to Gonze et al. [35]: 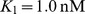, 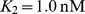, 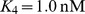, 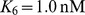, 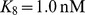, 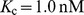, 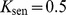, 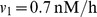, 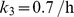, 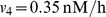, 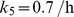, 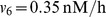, 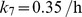, 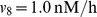. Furthermore, we introduce cell-to-cell variability in terms of different individual periods between cells in the same way as it was proposed by Gonze et al. [35]. The production and degradation rates 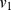, 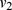, 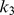, 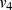, 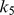, 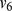, 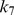 and 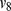 are divided by a scaling factor 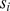, whose values are chosen randomly from a normal distribution of mean 1.0 and standard deviation 0.05.

## Results

**Figure 2:**
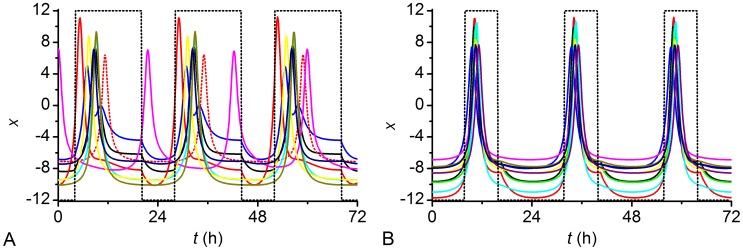
Single cell activity patterns of 10 randomly chosen neurons. (A) Summer conditions: 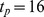, 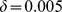. (B) Winter conditions: 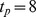, 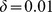. Note that the amplitude of the entraining signal is not in scale.

We entrained our network model over 40 cycles with a 24 h rhythm and variable photoperiod lengths. In all numerical calculations the initial conditions were randomly distributed around *x* = 1 and *y* = 0, according to a normal distribution with a standard deviation 0.2. In all simulations, the final dynamical state was attained after just a few periods (see Figure S1 in Text S1). Therefore, we chose to discard an initial transient of 15 cycles. In Figure 2 we show the time courses of activity in the *x*-coordinate of 10 randomly chosen cells for 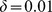 and 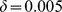. This parameter affects the number of long-range connections in the SCN network. It should be emphasized that the coupling of the cells in the network leads to an approximately 10-fold increase in the amplitude of the activity of the single cells, as observed also experimentally [21]. Moreover, it can be seen that decreasing 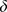 and thus the number of long-range connections leads to a broadening of the peak-phase distribution (see also Figure S2 in Text S1), whereas the activity peak of the individual cells always has a width of only about 4–5 h as observed experimentally [10].

**Figure 3:**
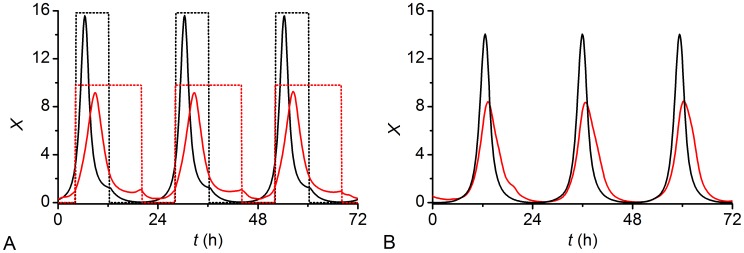
Global activity patterns of the SCN. Oscillatory profiles of the mean field for the entrained network (A) and for free-running conditions (B). In both panels red lines refer to summer conditions (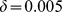), whereas black lines signify winter conditions (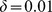). The amplitude of the entraining signal is not in scale.

To capture the effect of the broader phase distribution on the overall electrical activity of the SCN we calculated the mean activity over the entire SCN and shifted this activity into the positive quadrant (Figure 3A). It can be seen that the mean electrical activity is broader for small values of 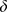, which we therefore refer to as “summer-topology" and is shorter for large values of 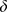, which we therefore refer to as “winter-topology". Therefore, our model is able to explain the different experimentally observed phase-distributions in summer and in winter [10], by changes in the network topology. It has also been observed that after entrainment to different photoperiods the width of the phase distribution is preserved under free-running conditions as well. In Figure 3B we show the network's dynamical response without an entraining stimulus for the winter- and summer-topology. We can observe that even without entraining the phase distribution is also preserved under free-running conditions. Thus we can conclude that the width of the activity is mainly related to SCN plasticity [9]. Moreover, even in the absence of light input the non-rhythmic cells in the VL region become rhythmic due to the coupling to the oscillators with a self-sustained rhythm in the DM region as already observed in a previous study on synchronization induced rhythmicity in the SCN [36]. Another surprising observation is that a coupling strength of 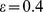 is sufficient to synchronize the oscillators, despite the significant heterogeneity in the oscillator periods. We attribute this to the weakness (small radial relaxation rate 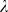) of the oscillators.

**Figure 4:**
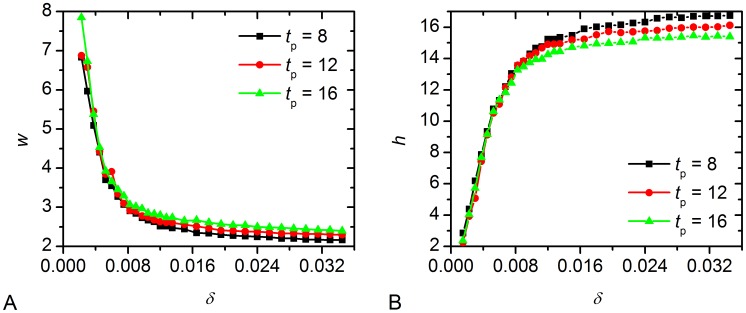
Analysis of the electrical activity of the SCN as a function of the network structure. The average width 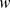 of the mean field signal (A) and its amplitude *h* (B) for different durations of the light signal. It can be observed that the shape of the signal depends predominately on the network structure. On the other hand, the duration of the light input only has an insignificant effect.

The sensitivity of the amplitude 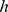 and width *w* (defined as the width of the peak at half the amplitude) of the mean electrical activity with respect to the parameter 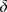 is shown in Figure 4. It can be seen that by changing the number of long-range connections the width of the mean electrical activity can be adjusted to winter and summer photoperiods. When moving from winter- to summer-topology also the mean activity amplitude decreases. To ensure that the width and amplitude of the mean activity is mainly determined by the network topology we also calculated them for short and long photoperiods. It can be seen that both only slightly depend on the length of the photoperiod (see Figure 4). Remarkably, the same conclusions can be drawn from results presented in Figure S3 in Text S1, where the mean-field of the summer (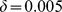) and winter topology (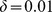) for different durations of the light input is shown.

**Figure 5:**
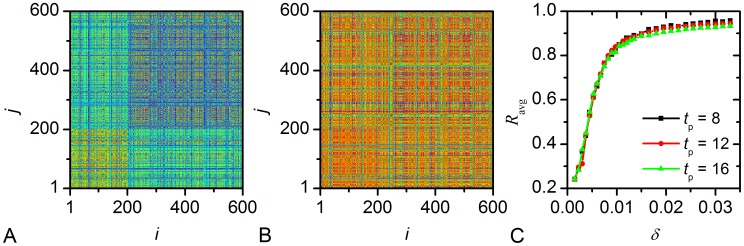
Correlations between individual SCN neurons. The correlation matrices 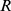 for summer (A) and winter conditions (B), and the average correlation coefficient 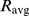 as a function of 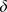 for different photoperiods (C). The color-profile of the values 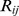 is linear between blue depicting 0 (no correlation) and red representing 1 (perfect correlation).

To quantify the synchronization between the neuronal cells we calculated the correlation matrices for the summer- and winter-topology under the corresponding entrainment cycle (Figure 5A–B). The *ij*-th element of the matrix is defined as follows:
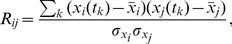
(11)where 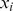 is one of the Cartesian coordinates which signifies the neuronal activity and 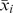 and 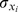 are the mean value and the standard deviation of the time series 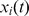 of the *i*-th oscillator, respectively. The sum in Eq. (11) runs over the whole temporal series, whereby every 25th point of 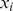 resulting from numerical integration is recorded. It can be seen that the cells receiving light input in the VL region are more synchronous to each other for both topologies. On the other hand, cells in the DM region in the summer-topology are asynchronous to each other reflecting the broader peak phase distribution, but are more synchronous to the cells in the VL region probably to the ones they have long-range connections to. For the winter-topology also the cells in the DM region show good synchrony to each other, which reflects the narrow phase distribution observed in short photoperiods [10]. The overall better synchrony with more long-range connections in the winter-topology also reflects itself in the average correlation coefficient 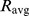 (Figure 5C). Besides, the level of correlation between cells only slightly depends on the duration of the photoperiod, hence once again confirming that the intercellular network structure is the key agent governing the characteristics of the collective neuronal activity.

**Figure 6:**
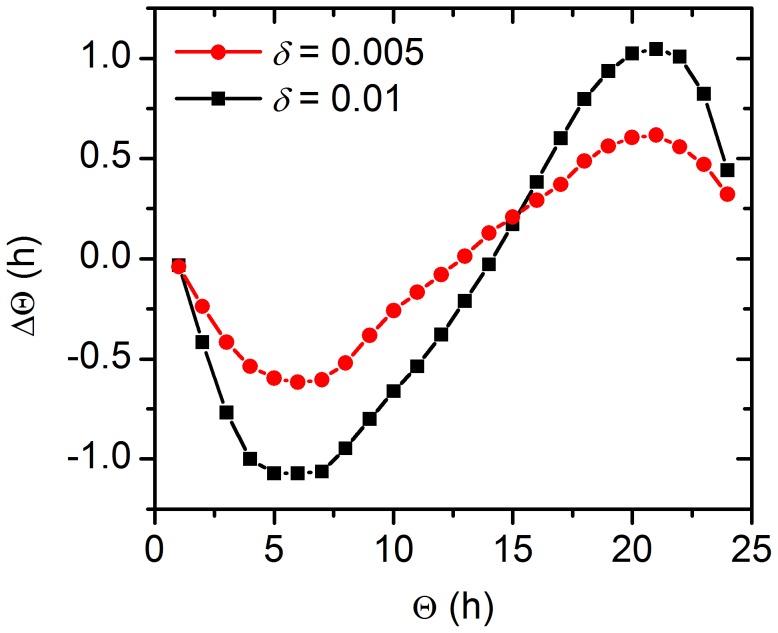
Phase response curves of the SCN network. Perturbation induced changes in the phase of the mean field signal for summer (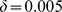) and winter (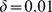) conditions. The applied pulse had a duration of 4 h and an amplitude of 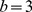.

**Figure 7:**
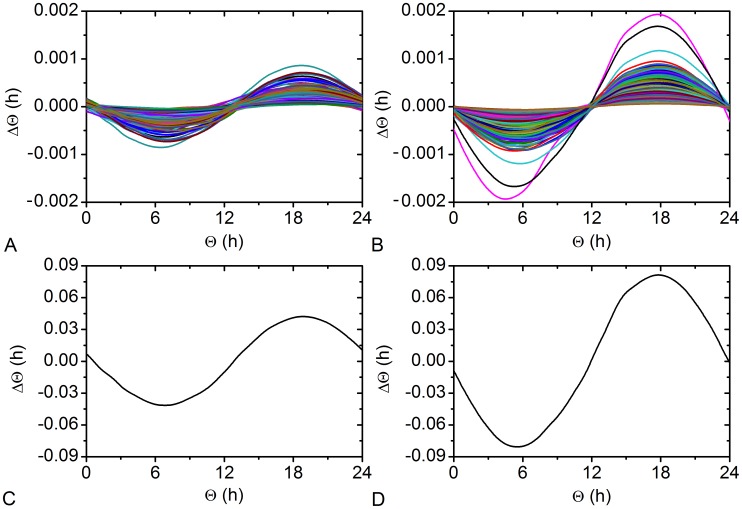
Instantaneous phase response curves of all light-receiving neurons. Individual PRCs for summer 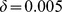 (A) and winter 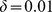 (B) conditions and the corresponding summed up phase responses (C) and (D). The unit of time is scaled to circadian time. The average correlation coefficient between the individual curves in the summer topology is 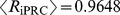, while for the winter topology it is 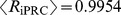.

Furthermore, it has been experimentally observed that the PRC of the SCN shows a larger magnitude in winter than in summer [63]. We have numerically calculated the PRC for the winter- and summer-topology (Figure 6). In accordance with the experimental observations, the PRC for the winter-topology has a higher magnitude then the summer-topology. Notably, the extent of the phase shift under both conditions is regulated by the amplitude of the perturbation signal (see Figure S4 in Text S1). To further analyze the underlying reasons for the differences, we calculated the individual instantaneous PRCs of all light-receiving oscillators (Figure 7A–B), which when summed up represent the overall instantaneous PRC (Figure 7C–D). It can be seen that the individual iPRCs are also diminished in magnitude in the summer topology compared to the winter topology. Moreover, the individual iPRCs are more shifted to each other in the summer topology (correlation coefficient 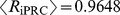 compared to 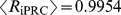), most likely due to the lesser synchronization between the individual light-receiving cells. Both effects lead to a diminished overall PRC. The effect due to shifting of the curves is enhanced for even smaller values of 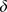: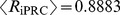 for 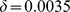 (Figure S5 in Text S1).

**Figure 8:**
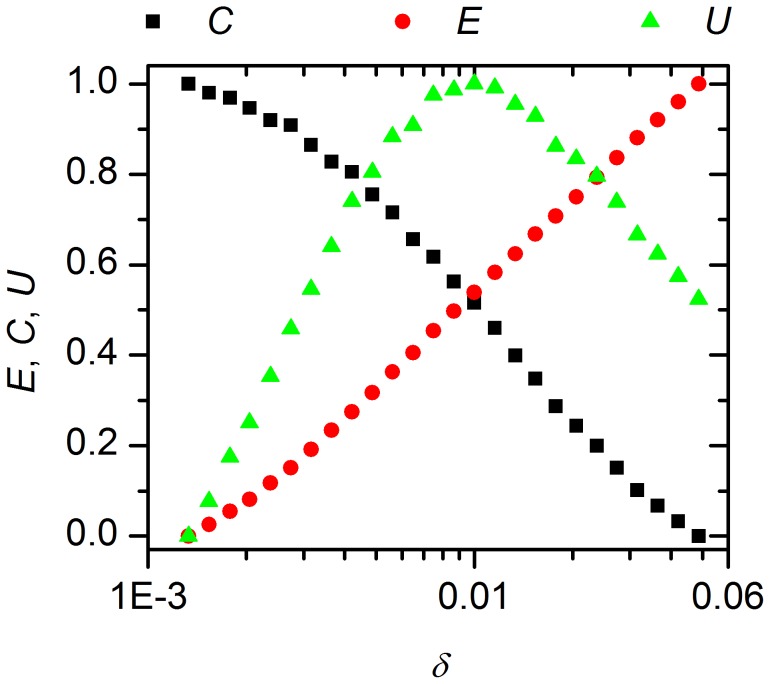
Characterization of the SCN network model. The global efficiency of the network 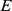, the clustering coefficient 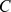 and their product 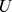 as a function of 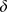. In the calculation 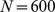 cells were considered, 1/3 of them being located in the ventrolateral (VL) region. Note that all quantities are scaled to the unit interval.

To characterize the different network topologies in winter and in summer we calculated the network efficiency and clustering coefficient, two properties that allow the characterization of small-world networks (Figure 8). It has been shown that the product of the two measures is the largest when the network possesses small-world properties 57,64. Using this measure characterizes the winter-topology as a small-world network, which is efficient in terms of costs for the neuronal connections and the achieved synchrony between the individual cells. On the other hand the summer-topology is not able to achieve synchrony due to the reduced number of long-range connections, which lessens the small-world properties of the network.

**Figure 9:**
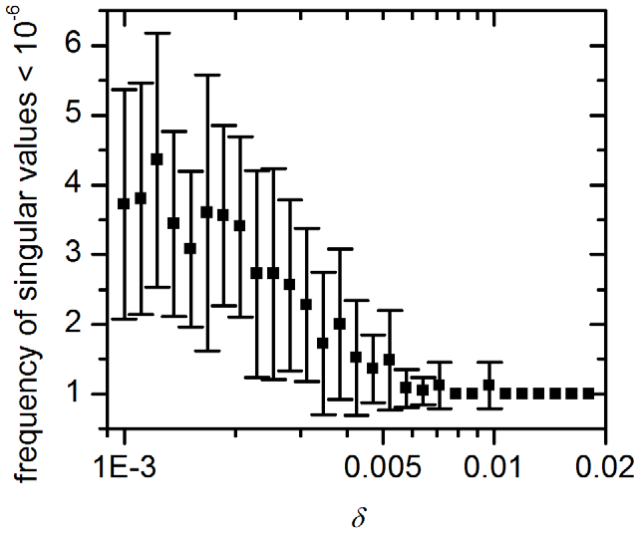
Spectral graph analysis for the detection of communities in the SCN network. Mean number of near zero singular values (<10^−6^) in the Laplacian matrix of the SCN network as a function of 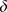. Presented are averages and standard deviations over 25 independent replicates.

To additionally characterize the synchronization behavior of the network we calculated the singular value spectrum of the network's Laplacian matrix ***L*** for 25 replicates of our network structure (see Section 5 and Figure S6 in Text S1). For a symmetric coupling it has been shown that large eigenvalues indicate groups of oscillator that synchronize fast, whereas near zero eigenvalues indicate a community structure inside the network, with groups of oscillators that do not synchronize their phases to each other [59], [60]. In Sections 2, 3 and 4 in Text S1 we generalize these numerical findings to a network of weakly coupled heterogeneous oscillators and show analytically that for a non-symmetric coupling small near-zero singular values lead to a large variance in the synchronized phase distribution. Although, the theory is established for a network of only self-sustained oscillators the numerical calculations indicate that its predicted effects are still applicable for the here considered network of damped and self-sustained oscillators. The singular value spectra for the summer topology possesses many near-zero eigenvalues indicating that indeed a community structure is present, where oscillators within a community are more connected to each other than to members from other communities (see Section 5 in Text S1). Our theoretical results now also explain how this effects the stable distribution of phases. In particular the near-zero eigenvalues present for the summer topology lead to a dramatic increase in the width of the phase distribution. To illustrate this even more we have also calculated the mean number of near zero singular values over different values of 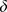 (Figure 9). It can be seen that for 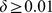 only the trivial zero singularvalue exists, whereas at lower values of 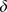 small non-zero singularvalues indicate groups of oscillators that do not fully synchronize their phases to each other and therefore lead to a broader SCN activity.

**Figure 10:**
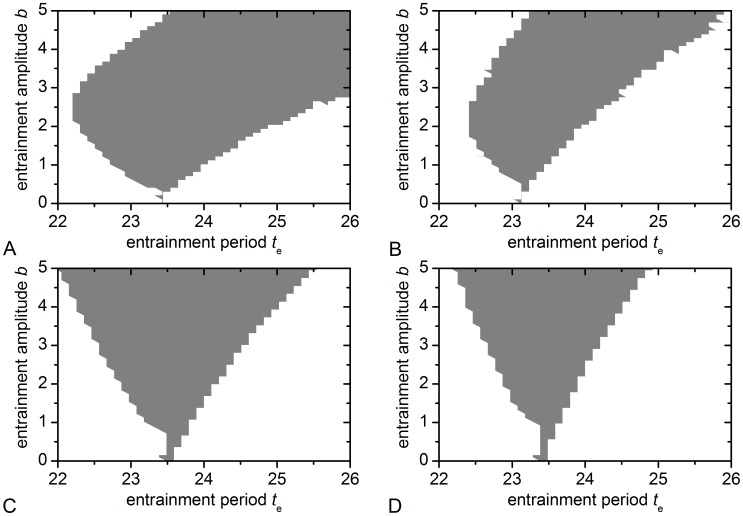
Entrainment regions of the oscillator network. The grey shaded region depicts combinations of the entrainment period 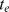 and entrainment amplitude *b* that allow entrainment to the external signal. (A) Winter conditions: 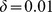 and (B) summer conditions: 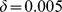 with coupling strength 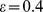. (C) Winter conditions and (D) summer conditions but the coupling strength was raised to 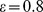.

To further characterize our model we also analyzed the entrainment properties of our model. In Section 5 in Text S1 we analyze the entrainment dynamics of a single, uncoupled oscillator in an analytic way and derive, several explicit formulae for the entrainment borders (see also Figure S7 in Text S1). These results shows that the individual weak, spiking single oscillator we consider shows an entrainment behavior that markedly differs from the entrainment of a rigid amplitude-phase oscillator. Especially, after a certain threshold of the entrainment amplitude *b* the external forcing overrides the internal dynamics and the oscillator is practically entrainable to every period 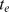. We therefore compared the entrainment region of the single uncoupled oscillator (Figure S7 in Text S1, dotted lines) with the entrainment region of the oscillator network in summer and winter (Figure 10A–B). Note that due to the high computational costs, we considered only one network realization per condition. Therefore, we expect that the borders of the entrainment region slightly change with each realization. Nevertheless, even the calculations for just one realization give valuable insights. We find that due to the coupling the oscillator network behaves more like a rigid oscillator as found also in a previous study [44]. Moreover, the entrainment amplitude *b* needs to be significantly higher compared to the single oscillator to achieve entrainment. This effect is related to the amplitude expansion of the coupled oscillators. This is confirmed by calculations of the entrainment region for an oscillator network with increased coupling strength 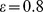 (Figure 10C–D). The entrainment region becomes even smaller due to increased rigidity and amplitude of the oscillator network in line with the previous results [44].

Nevertheless, we observe that the entrainment region of the winter topology is larger than that of the summer topology. This result is in line with the observed reduced phase response curve of the summer topology (Figure 6). Another interesting observation is that for a small coupling strength the entrainment region is not symmetric for large entrainment amplitudes and especially, entrainment to smaller periods is not possible.

In order to verify that our findings are indeed qualitatively independent of the system size we calculated how the amplitude 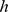 and the width 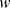 of the mean electrical activity change with respect to the parameter delta for a three times larger system size (1800 neurons, 600 of them being located in the VL region). First, we computed at which values of delta small-world network properties are obtained. Results shown in Figures S8A–B in Text S1 reveal that those characteristics, which reflect winter conditions, are found around 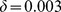. Accordingly, the SCN network structure reflecting summer conditions was chosen to be at 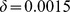. Remarkably, the results in Figures S8C–D in Text S1, showing the changes of *w* and *h* as a function of delta, clearly indicate that qualitative identical results are obtained for larger system sizes as well (compare to Figure 4).

**Figure 11:**
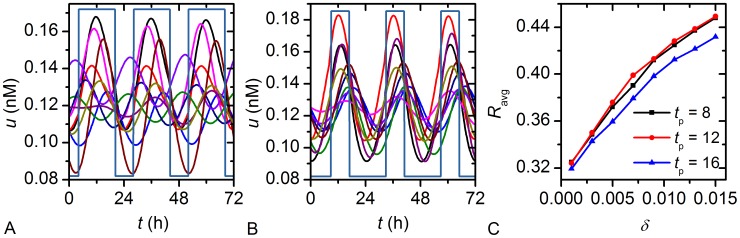
Single cell activity patterns from 10 randomly chosen cells driven by the Goodwin model. (A) summer conditions: 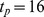, 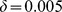 and (B) winter conditions: 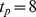, 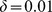, and (C) the corresponding average correlation coefficient 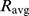 as a function of 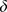 for different photoperiods (C). Note that in panels (A) and (B) the amplitude of the entraining signal is not in scale.

Finally, we examine how the SCN network behaves in the case that a more complex and biologically relevant model – the Goodwin oscillator – drives the dynamics of individual cells. Results presented in Figures 11A–B show the time evolution of the concentration of the clock gene *u* in summer (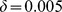) and winter (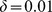) conditions. Obviously, the phase distribution is much broader in summer conditions than in winter conditions. This observation is additionally confirmed with the results presented in Figure S9 in Text S1, where the time evolution of the mean SCN activity over several entraining cycles is shown. Similar as in the case of the simple amplitude-phase oscillator (see Figure 3), the mean field signal of the Goodwin oscillator network is higher and narrower in winter. Furthermore, Figure 11C shows the average correlation coefficient as a function of the network parameter, whereby a greater extent of synchronization can be observed as 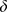 is increased. Thus, the results obtained with the more complex model for circadian oscillations are qualitatively very similar to those obtained with the simple amplitude-phase oscillator.

## Discussion

The importance of network plasticity for the adaptation of the circadian rhythm to different photoperiods was put forth in several experimental studies [9], [10]. Using our model, we identified one possible mechanism able to explain the adaptation to different photoperiods: the introduction of long-range connections between cells in the VL (ventrolateral) and DM (dorsomedial) region of the SCN. In particular, the length of the behavioral activity can be regulated as follows: dense long-range connections during winter lead to a narrow activity phase, while rare long-range connections during summer lead to a broad activity phase. We found that this result is independent of the number of neurons. Moreover, similar results were obtained when replacing the model governing the dynamics of the individual neurons, namely the simple amplitude-phase oscillator, with the more biologically relevant Goodwin oscillator. To go from a wide phase distribution in summer to a more narrow and synchronized phase distribution in winter roughly the doubled number of long-range connections had to be introduced. In our model of 600 neurons, this amounts to around 450 connections in the summer topology and 900 connections in the winter topology. Taking into account that the fraction of added long range connections 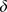 leading to a network with small world properties is inversely proportional to the number of oscillators [57], [58], we can extrapolate the number of required long-range connections in the real SCN network consisting of 20000 neurons. In this case, roughly 15000 connections would need to be introduced to change the network from the summer-topology to the winter-topology.

It should be emphasized that the photoperiod gradually changes from summer to winter and hence the introduction of the additional neuronal connections would occur in a time span over roughly half a year. In fact the physical connections between neurons do not need to be introduced but synaptic plasticity changing the responsiveness of individual neurons could lead to a weakening or strengthening of connections. To support those ideas we also calculated how the dynamics of the SCN network changes if the coupling strength of the long-range connections in a winter topology network with 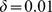 is gradually reduced. The simulation results indicate that reducing the coupling strength by at least 75% for half of the long-range connections (reflecting the summer topology with 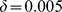) leads to very similar effects as a complete deletion of links (Figure S10 in Text S1). Moreover, a simulation with a rapid transition from winter to summer topology shows that the entrainment transient is very short and therefore negligible for gradual changes over the year (Figure S11 in Text S1).

Our model is also able to simulate the increased magnitude of the overall phase response curve (PRC) in winter compared to summer. This can be explained by the reduced magnitude of the individual instantaneous PRCs and their shifting relatively to each other in the summer topology, leading to an overall smaller phase response. Both effects are due to the reduced synchrony in the summer-topology shown by the correlation matrices in Figure 5. Since the cells are at different phases of their inner clock when not completely synchronized this leads to a smaller overall response, much like is the case for coupled but unsynchronized pendulums that are perturbed by a pulse. Of course, the individual PRCs do not contain a “dead" zone with no phase shift. The reason for this is the simple model for the individual oscillators, which cannot account for the complicated phase response behavior of more realistic models. However, the focus of this work was not on modeling the individual oscillators but analyzing the impact of network changes on the synchrony of the overall SCN and how photoperiod adaptation is related to this.

Along with the phase response, we also analyzed the dependence of the entrainment region of the oscillator network on the network topology and the coupling strength between the individual neurons. Our results agree with previous studies that found a decreased entrainment region for increased coupling between the oscillators, due to an increased in rigidity and amplitude of the oscillators [44]. Moreover, in line with the observed reduced PRC of the summer topology we also observe that the entrainment region of the summer topology is smaller. This result is counterintuitive, since intuitively one would expect that the oscillators become more rigid in a network with more long-range connections. In addition, the amplitude of the oscillator networks mean field is larger in the winter topology as compared to the summer topology. A common statement in the analysis of oscillator synchronization is that large amplitude oscillators are harder to entrain. Our results show that this statement cannot simply be carried over to oscillator networks. It seems that the effect of adding specific connections between cells is different from an increase in the overall coupling strength. Whereas, the latter leads to a decrease in the entrainment region, due to amplitude expansion and increase in rigidity, the former leads to a larger entrainment region due to a more synchronized phase response. These findings underline the importance of the network structure connecting the oscillators.

Another possibility to achieve different phase distributions in summer and winter are changes in the intrinsic oscillator characteristics or in the transmission of signals between the cells. For example the introduction of a delay distribution in the synaptic transmission also leads to a distribution of peak phases [32]. Changing the delay distribution could also allow the adaptation to different photoperiods. However, it is difficult to explain a delay of several hours physically since it has been observed that the transcriptional induction of *Per* upon a light pulse is within 5–15 minutes during subjective night [13]. Since the same pathway induces transcription in cells not sensing light but neuropeptide release, we can assume that the delay times are on the same order of magnitude also for intercellular coupling via neuropeptides.

Our results support the ideas pointed out by Meijer et al. [10] in that the SCN neuronal network plasticity crucially affects the activity phase distribution among SCN neurons and therefore contributes to the adaptation to changes in day length. Interestingly, our results indicate that the intercellular communication network in the SCN has features of a small-world network. Such complex topologies have been identified in numerous biological systems including functional as well as anatomical connections in the nervous system [65]. They indeed seem to be advantageous for various living organisms. A closer inspection of Figures 4A–B and 5 reveals, that below the region where the small-world properties of the network are well expressed (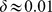, see Figure 8) the curves are very steep. For the SCN it would thus be advantageous operating in the proximity of the optimal small-world configuration. Namely, rather small modifications in the extent of long-range connections enable the regulation of synchronization behavior and phase distributions of electrical activity in the neuronal population. In this manner the plasticity as well as the realization of different neuronal coupling mechanisms between the ventral and dorsal SCN have a large impact during the adjustment to seasonal changes.

The effect of the broad phase-distribution in the summer topology is explained by the introduction of well-connected communities separated by bottlenecks into the network. Local clusters of SCN oscillator synchronize fast and well to each other. However, the synchronization between these clusters is hindered due to the few connections between them. The community structure of a network can be quantified by the so called algebraic connectivity given by the second smallest eigenvalue of the networks Laplacian matrix (see Eq. 5) [66], [67]. If this eigenvalue is near zero the network can be easily separated into groups. It was shown numerically that near-zero eigenvalues lead to a more unsynchronized state of the oscillators [60], [68]. Our analytical results (Sections 2, 3 and 4 in Text S1) support these findings and show that the distribution variance of oscillator phases is strongly influenced by near-zero eigenvalues. Remarkably, these theoretical results do not depend on the underlying model governing the dynamics of individual cells and therefore generalize the proposed mechanism of photoperiod adaptation by controlling the number of long-range connections and consequently the community structure in the SCN network. Another advantage of the theoretical results is the insight into the relation between synchronized phase and period distribution on the one hand and network properties on the other hand (see for example Eq. 18 in Text S1). Future studies could use this relation to extend our model to account for other features of the phase distribution in the SCN, for example the observed bimodal phase distribution in long photoperiods [30], [31].

## Supporting Information

Text S1 The supporting Text S1 provides several theoretical definitions and derivations generalizing the findings of the main study. In Section 1 the concept of phase response curves is introduced and it is shown how to compute them numerically. In Section 2 a general equation for the dynamics of the in-phase distribution of an arbitrary oscillator network with small heterogeneity between the oscillators is derived. The derivation is based on the well-known phase reduction method introduced by Kuramoto and a linearization around small phase differences. In Section 3 we derive the stationary phase-distribution and the locked frequency using the pseudoinverse of the matrix M of the linearized system. Moreover, we show that the stationary phase distribution is among other factors mostly influenced by near-zero singular/eigenvalues of the matrix M. In Section 4 we consider several special cases of oscillator coupling. In particular we derive that for a coupling function that is the same for all oscillators the networks Laplacian and consequently its near-zero singular/eigenvalues determine the stationary phase-distribution. In Section 5 we connect the findings from Section 2–4 to previous work from spectral graph theory, showing that the network structure, and in particular the occurrence of communities with nodes that are well connected within the community but weakly connected between communities, is tightly related to the singular/eigenvalue spectrum of the Laplacian. Moreover, we calculate and discuss the spectra for the winter and summer topology of our SCN network. In Section 6 we additionally analyze theoretically the entrainment to an external stimulus of a single amplitude-phase oscillator from our study. We derive novel entrainment bounds for several special cases of rigid and weak oscillators. These bounds are derived to compare them against the entrainment of the whole SCN network. Section 7 contains all supplementary figures along with figure captions of our study.
(PDF)
