# Supplementary material for: Modeling the Seasonal Adaptation of Circadian Clocks by Changes in the Network Structure of the Suprachiasmatic Nucleus
Source: PLoS Comput Biol. 2012 Sep 20;8(9):e1002697. doi: 10.1371/journal.pcbi.1002697 (PMC3447953; doi:10.1371/journal.pcbi.1002697)
Supplement: Text S1 — The supporting Text S1 provides several theoretical definitions and derivations generalizing the findings of the main study. In Section 1 the concept of phase response curves is introduced and it is shown how to compute them numerically. In Section 2 a general equation for the dynamics of the in-phase distribution of an arbitrary oscillator network with small heterogeneity between the oscillators is derived. The derivation is based on the well-known phase reduction method introduced by Kuramoto and a linearization around small phase differences. In Section 3 we derive the stationary phase-distribution and the locked frequency using the pseudoinverse of the matrix M of the linearized system. Moreover, we show that the stationary phase distribution is among other factors mostly influenced by near-zero singular/eigenvalues of the matrix M. In Section 4 we consider several special cases of oscillator coupling. In particular we derive that for a coupling function that is the same for all oscillators the networks Laplacian and consequently its near-zero singular/eigenvalues determine the stationary phase-distribution. In Section 5 we connect the findings from Section 2–4 to previous work from spectral graph theory, showing that the network structure, and in particular the occurrence of communities with nodes that are well connected within the community but weakly connected between communities, is tightly related to the singular/eigenvalue spectrum of the Laplacian. Moreover, we calculate and discuss the spectra for the winter and summer topology of our SCN network. In Section 6 we additionally analyze theoretically the entrainment to an external stimulus of a single amplitude-phase oscillator from our study. We derive novel entrainment bounds for several special cases of rigid and weak oscillators. These bounds are derived to compare them against the entrainment of the whole SCN network. Section 7 contains all supplementary figures along with figure captions of our study. [file pcbi.1002697.s001.pdf]

Modeling the Seasonal Adaptation of Circadian Clocks by changes in the Network Structure of the Suprachiasmatic Nucleus

Christian Bodenstein, Marko Gosak, Stefan Schuster, Marko Marhl, Matjaž Perc

Supplementary online information

Contents

1. Instantaneous phase response curves (iPRCs) 2

2. Phase synchronization of weakly coupled oscillator networks 3

3. Synchronized frequency and its phase distribution 5

4. Special cases of coupling 7

5. Spectral Graph Analysis and Synchronization 8

6. Entrainment of a single amplitude-phase oscillator 10

7. Supplementary Figures S1-S11 16

References 22

1. Instantaneous phase response curves (iPRCs)

The network of N connected oscillator cells in our model can be described by the generalized ordinary differential equation:

$$\dot{\boldsymbol{X}} = \boldsymbol{F}(\boldsymbol{X}). \quad (1)$$

If the coupling between the cells is strong enough, this equation possesses a limit cycle solution $\boldsymbol{X}^0(t) = \boldsymbol{X}^0(t+\tau)$ with the period τ. A light pulse subjected to N_l of the oscillator network along a specific dimension d can be described as a perturbation of the above equation:

$$\dot{\boldsymbol{X}} = \boldsymbol{F}(\boldsymbol{X}) + \boldsymbol{L}bP(t), \quad (2)$$

where $\boldsymbol{L}$ is a diagonal matrix that determines which oscillators (and oscillator dimension) are subjected to the external light-input $P(t)$ with amplitude b. If the external perturbation amplitude b is small compared to the amplitude of the limit cycle, it is valid to reduce the dynamics to the perturbation of the phase of the limit cycle solution [1]. The equation describing phase dynamics is given by:

$$\dot{\phi} = 1 + b\sum_{j=1}^{N_l} \mathrm{iPRC}_j(\phi)\, P(t). \quad (3)$$

The function $\mathrm{iPRC}_j(\phi) = Q_{j,d}(\phi)$ describes the instantaneous phase response along the perturbed dimension d of the jth oscillator [2, 3]. It can be calculated by integrating the following equation backwards in time from a given reference point on the limit cycle [3, 4]:

$$\dot{\boldsymbol{Q}}(t) = -\boldsymbol{J}(t)\boldsymbol{Q}(t), t \le t_f. \quad (4)$$

The final condition is $\boldsymbol{Q}(t_f) = \left(0, \dots, 0, 1/F_l\left(\boldsymbol{X}^0(t_f)\right), 0, \dots, 0\right)^T$, where the entry $1/F_l\left(\boldsymbol{X}^0(t_f)\right)$ is at the lth position and ensures a normalization such that $\boldsymbol{Q}(t) \cdot \boldsymbol{F}(\boldsymbol{x}(t)) = 1$. The matrix $\boldsymbol{J}(t)$ is the Jacobian matrix evaluated along the limit cycle. As an example we computed the iPRCs for the oscillators in our SCN network with the parameter δ determining the number of long-range connections set to 0.0035 (Section 7, Figure S5). For a square pulse $bP(t)$ with short duration during the phases ϕ_1 and ϕ_2 and small amplitude b, the iPRCs can be used to approximate the PRC [2]:

$$\mathrm{PRC}(\phi) = -b\int_{\phi_1}^{\phi_2} \sum_{j=1}^{N_l} \mathrm{iPRC}_j(\phi)\, d\phi. \quad (5)$$

This shows that the magnitude and shape of the overall PRC is determined by the sum of the iPRCs of the individual oscillators.

2. Phase synchronization of weakly coupled oscillator networks

It is known that N uncoupled identical oscillators $\boldsymbol{x}_1 = \boldsymbol{x}_2 = \cdots = \boldsymbol{x}_N = \boldsymbol{x}$ possess N zero Floquet exponents that correspond to perturbations in the direction of the phase. Weak coupling of the oscillators (small coupling strength ε) leads to $N-1$ of these Floquet exponents becoming ε-small. These exponents determine the rate of relaxation to synchronization in the subspace of phases of the oscillator network. The Floquet exponents $\varepsilon\lambda_i$ can be approximated by the eigenvalues λ_i of the following matrix [5]:

$$\boldsymbol{M} = \begin{pmatrix} -\sum_i^{i\neq 1} \kappa_{1i} & \cdots & \kappa_{1N} \\ \vdots & \ddots & \vdots \\ \kappa_{N1} & \cdots & -\sum_i^{i\neq N} \kappa_{Ni} \end{pmatrix}, \tag{6}$$

where:

$$\kappa_{ij} = \frac{1}{\tau}\int_0^\tau \boldsymbol{Q}(t)\frac{\partial \boldsymbol{C}_i}{\partial x_j}(\boldsymbol{x}(t))\boldsymbol{F}(\boldsymbol{x}(t))\,dt. \tag{7}$$

Here $\boldsymbol{Q}(t)$ is the vector of iPRCs (see eq. 4) along each dimension of the uncoupled oscillator $\boldsymbol{x}(t)$ and $\boldsymbol{F}(\boldsymbol{x}(t))$ is the right-hand side of the uncoupled oscillator evaluated along the limit-cycle. The Jacobian matrix $\frac{\partial \boldsymbol{C}_i}{\partial \boldsymbol{x}_j}$ describes how the dimensions of the oscillators i and j are coupled to each other along the limit cycle.

In fact this result can be proven for a more general setup by using the phase reduction method [1]. Consider the network of coupled heterogeneous n-dimensional oscillators:

$$\dot{\boldsymbol{x}}_i = \boldsymbol{F}_i(\boldsymbol{x}_i, \varepsilon, \boldsymbol{x}_1, \dots, \boldsymbol{x}_N),\ i = 1, \dots, N, \tag{8}$$

where we can expand the right-hand side for small ε as $\boldsymbol{F}_i(\boldsymbol{x}_i, \varepsilon, \boldsymbol{x}_1, \dots, \boldsymbol{x}_N) = \boldsymbol{F}_i(\boldsymbol{x}_i) + \varepsilon\frac{\partial \boldsymbol{F}_i}{\partial \varepsilon}(\varepsilon = 0, \boldsymbol{x}_1, \dots, \boldsymbol{x}_N)$. Thus, we can write the coupled system as:

$$\dot{\boldsymbol{x}}_i = \boldsymbol{F}_i(\boldsymbol{x}_i) + \varepsilon\boldsymbol{C}_i(\boldsymbol{x}_1, \dots, \boldsymbol{x}_N),\ i = 1, \dots, N. \tag{9}$$

Further we assume that the differences in the oscillators are small and, for simplicity, of the same order of magnitude as the coupling strength ε. This implies that the deviations in the oscillator frequencies ω_i are also ε-small. The imaginary oscillator derived after averaging over the uncoupled oscillators has a limit cycle solution $\boldsymbol{x}^0(t) = \boldsymbol{x}^0(t + 2\pi/\langle\omega\rangle)$, with average period $\tau = 2\pi/\langle\omega\rangle$, $\langle\omega\rangle = 1/N\sum_i \omega_i$, and is governed by the averaged right hand side $\boldsymbol{F}(\boldsymbol{x})$. $\boldsymbol{Q}(t)$ is the vector of iPRCs of the uncoupled imaginary average oscillator and is τ periodic. To avoid complicated expressions it is convenient to redefine $\boldsymbol{x}^0$ and $\boldsymbol{Q}$ as 2π periodic functions: $\boldsymbol{x}^0(t) := \boldsymbol{x}^0(1/\langle\omega\rangle\, t)$, $\boldsymbol{Q}(t) := \boldsymbol{Q}(1/\langle\omega\rangle\, t)$.

The reduction to the individual oscillator phases $0 \leq \phi_i \leq 2\pi$ yields [1]:

$$\dot{\phi}_i = \omega_i + \varepsilon\boldsymbol{Q}(\phi_i)\boldsymbol{C}_i\big(\boldsymbol{x}^0(\phi_1), \dots, \boldsymbol{x}^0(\phi_N)\big). \tag{10}$$

We now define the phase differences to the synchronized state: $\psi_i = \phi_i - \Omega t$ with $-2\pi \leq \psi_i \leq 2\pi$. The frequency of the phase locked state, Ω, is determined by a solvability condition for the stationary phase differences. It will be given in the next section. The evolution of the phase differences is then governed by:

$$\dot{\psi}_i = \omega_i - \Omega + \varepsilon \boldsymbol{Q}(\Omega t + \psi_i)\boldsymbol{C}_i\big(\boldsymbol{x}^0(\Omega t + \psi_1), \dots, \boldsymbol{x}^0(\Omega t + \psi_N)\big). \tag{11}$$

Here we assume that the differences $\varepsilon\Delta\omega_i = \omega_i - \Omega$ of the individual frequencies to the synchronized frequency are ε-small. We will see that this is justified if the differences to the average oscillator frequency are ε-small, which we assumed above. Then we can write:

$$\dot{\psi}_i = \varepsilon\Big(\Delta\omega_i + \boldsymbol{Q}(\Omega t + \psi_i)\boldsymbol{C}_i\big(\boldsymbol{x}^0(\Omega t + \psi_1), \dots, \boldsymbol{x}^0(\Omega t + \psi_N)\big)\Big). \tag{12}$$

Since ε is small the changes in the ψ_i are slow compared to the movement of the reference oscillator. Therefore, we are permitted to average over one cycle $\tau_\Omega = 2\pi/\Omega$ and consider ψ_i on the slow time scale εt:

$$\dot{\psi}_i = \Delta\omega_i + \frac{1}{\tau_\Omega}\int_0^{\tau_\Omega} \boldsymbol{Q}(\Omega t + \psi_i)\boldsymbol{C}_i\big(\boldsymbol{x}^0(\Omega t + \psi_1), \dots, \boldsymbol{x}^0(\Omega t + \psi_N)\big)\, dt. \tag{13}$$

The integral term is only a function of the phase differences $\psi_{ij} = \psi_j - \psi_i$. This can be seen by rewriting the term as:

$$\frac{1}{\tau_\Omega}\int_0^{\tau_\Omega} \boldsymbol{Q}(\Omega t + \psi_i)\boldsymbol{C}_i\big(\boldsymbol{x}^0(\Omega t + \psi_i + \psi_1 - \psi_i), \dots, \boldsymbol{x}^0(\Omega t + \psi_i + \psi_N - \psi_i)\big)\, dt$$

$$= \Gamma_i(\psi_1 - \psi_i, \dots, \psi_N - \psi_i). \tag{14}$$

Due to the in-phase locking of the oscillators we can linearize the term around small phase differences and finally obtain:

$$\dot{\psi}_i = \Delta\omega_i + \Gamma_i(\boldsymbol{0}) + \sum_{j=1}^{N} \frac{1}{2\pi}\int_0^{2\pi} \boldsymbol{Q}(t)\frac{\partial \boldsymbol{C}_i}{\partial x_j}\big(\boldsymbol{x}^0(t)\big)\boldsymbol{F}\big(\boldsymbol{x}^0(t)\big)\, dt\,(\psi_j - \psi_i), \tag{15}$$

where $\Gamma_i(\boldsymbol{0}) = \frac{1}{2\pi}\int_0^{2\pi} \boldsymbol{Q}(t)\boldsymbol{C}_i\big(\boldsymbol{x}^0(t)\big)\, dt$ is the contribution to the i-th oscillators frequency arising due to the coupling to the other oscillators. We can write the system in matrix form using the matrix from eq. 6:

$$\dot{\boldsymbol{\psi}} = \boldsymbol{\Delta\widetilde{\omega}} + \boldsymbol{M\psi}, \tag{16}$$

with $\boldsymbol{\Delta\widetilde{\omega}} = (\Delta\omega_1 + \Gamma_1(\boldsymbol{0}), \dots, \Delta\omega_N + \Gamma_N(\boldsymbol{0}))^T$.

A phase distribution $\boldsymbol{\psi}^0$ around the synchronized frequency phase is given by solving the linear equation system $\boldsymbol{\Delta\widetilde{\omega}} + \boldsymbol{M\psi}^0 = 0$ and we will consider it in more detail in the next section. The stability and time scale of approaching this phase distribution is given by the

eigenvalues of the matrix $\boldsymbol{M}$. Transforming the system into normal coordinates (denoted by α), given by the eigenvectors of the matrix $\boldsymbol{M}$, makes this more obvious [6]:

$$\dot{\boldsymbol{\psi}}^{\alpha} = \boldsymbol{\Delta}\widetilde{\boldsymbol{\omega}}^{\alpha} + \mathrm{diag}(\lambda_1, \dots, \lambda_N)\boldsymbol{\psi}^{\alpha}. \tag{17}$$

Note that some of the eigenvalues might also have an algebraic and/or geometric multiplicity, which we did not take into account here. If all eigenvalues of $\boldsymbol{M}$ have a negative real part, the synchronized phase distribution $\boldsymbol{\psi}^0$ is stable and all oscillators are locked in-phase with frequency Ω.

3. Synchronized frequency and its phase distribution

It was shown that the magnitude of the Floquet exponents determines how fast the oscillators or groups of oscillators synchronize their phases to each other [7, 8]. Especially, near-zero eigenvalues indicate a clustering in the network with groups of oscillators that do not or only weakly synchronize to each other. It was numerically shown that the phase-of-peak distribution $\boldsymbol{\psi}^0$ gets broader the more near-zero eigenvalues the Laplacian of the network possesses and consequently the more clustered the network becomes. Here, we present an analytical derivation of this for the general case considered above.

The synchronized phase distribution $\boldsymbol{\psi}^0$ is given by solving the linear equation system:

$$\boldsymbol{M}\boldsymbol{\psi}^0 = -\boldsymbol{\Delta}\widetilde{\boldsymbol{\omega}}. \tag{18}$$

A simple inverse of the matrix $\boldsymbol{M}$ does not exist, because one of its eigenvalues is zero: $\lambda_1 = 0$ with corresponding eigenvector $\boldsymbol{v}_1 = 1/\sqrt{N}\,(1, \dots ,1)^T$. This means that the system is neutrally stable to a simultaneous shift in all phases. This is to be expected since we can view the overall network as an oscillator, which again has a zero eigenvalue into the direction of its phase.

A solution to eq. 18 can be given in terms of the pseudoinverse $\boldsymbol{M}^+$ of $\boldsymbol{M}$:

$$\boldsymbol{\psi}^0 = -\boldsymbol{M}^+\boldsymbol{\Delta}\widetilde{\boldsymbol{\omega}} + (\boldsymbol{I} - \boldsymbol{M}^+\boldsymbol{M})\boldsymbol{w}, \tag{19}$$

with $\boldsymbol{w}$ chosen arbitrary. The pseudoinverse can be given in terms of a singular value decomposition (SVD) of $\boldsymbol{M}$:

$$\boldsymbol{M} = \boldsymbol{U}\boldsymbol{\Sigma}\boldsymbol{V}^T,\ \boldsymbol{M}^+ = \boldsymbol{V}\boldsymbol{\Sigma}^+\boldsymbol{U}^T. \tag{20}$$

Here $\boldsymbol{U}$ and $\boldsymbol{V}$ are orthonormal matrices with columns made up by the left and right singular vectors, respectively. In our case the kernel vector $\boldsymbol{v}_1 = 1/\sqrt{N}\,(1, \dots ,1)^T$ of $\boldsymbol{M}$ is one of the singular vectors of $\boldsymbol{V}$ and $\boldsymbol{u}_1$ the kernel vector of $\boldsymbol{M}^T$ is one of the singular vectors of $\boldsymbol{U}$. $\boldsymbol{\Sigma}$ is a diagonal matrix with the singular values $\sqrt{\sigma_i}$ corresponding to the singular vectors along the

diagonal. The singular values are the square roots of the eigenvalues of $\boldsymbol{MM}^T$ or $\boldsymbol{M}^T\boldsymbol{M}$ and any zero eigenvalue is also a singular value. For convenience we put the zero singular value σ_1 into the upper left corner of $\boldsymbol{\Sigma}$. The pseudoinverse of a diagonal matrix is simply given by taking the reciprocal of each non-zero element on the diagonal, leaving the zeros in place, and transposing the resulting matrix.

The linear equation system in eq. 18 is only consistent and thus has at least one exact solution, when the following condition holds:

$$\boldsymbol{MM}^+\boldsymbol{\Delta\widetilde{\omega}} = \boldsymbol{\Delta\widetilde{\omega}} = (\omega_1\varepsilon^{-1} + \Gamma_1(\boldsymbol{0}) - \Omega\varepsilon^{-1}, \ldots, \omega_N\varepsilon^{-1} + \Gamma_N(\boldsymbol{0}) - \Omega\varepsilon^{-1})^T. \tag{21}$$

If this equation does not hold the solution in eq. 19 will only be a least fit to eq. 18 and thus not a stable solution because then the right-hand side is not exactly zero in eq. 16. Using this condition we can determine Ω:

$$\boldsymbol{MM}^+ = \boldsymbol{U\Sigma}^+\boldsymbol{VV}^T\boldsymbol{\Sigma U}^T = \boldsymbol{U\Sigma\Sigma}^+\boldsymbol{U}^T$$

$$= \boldsymbol{U}\begin{pmatrix} 0 & 0 & \cdots & 0 & 0 \\ 0 & 1 & & 0 & 0 \\ \vdots & & \ddots & & \vdots \\ 0 & 0 & & 1 & 0 \\ 0 & 0 & \cdots & 0 & 1 \end{pmatrix}\boldsymbol{U}^T = \boldsymbol{I} - \boldsymbol{U}\begin{pmatrix} 1 & 0 & \cdots & 0 & 0 \\ 0 & 0 & & 0 & 0 \\ \vdots & & \ddots & & \vdots \\ 0 & 0 & & 0 & 0 \\ 0 & 0 & \cdots & 0 & 0 \end{pmatrix}\boldsymbol{U}^T = \boldsymbol{I} - \boldsymbol{u}_1\boldsymbol{u}_1^T.$$

Therefore, we obtain $\boldsymbol{u}_1\boldsymbol{u}_1^T\Omega(1, \ldots, 1)^T = \boldsymbol{u}_1\boldsymbol{u}_1^T\big(\omega_1 + \varepsilon\Gamma_1(\boldsymbol{0}), \ldots, \omega_N + \varepsilon\Gamma_N(\boldsymbol{0})\big)^T$ and finally:

$$\Omega = \frac{\sum_i u_{1i}(\omega_i + \varepsilon\Gamma_i(\boldsymbol{0}))}{\sum_i u_{1i}}. \tag{22}$$

Thus, besides the variations in the oscillators, the kernel vector of $\boldsymbol{M}^T$ determines the locked frequency. For the derivation to be closed, we now need to show that the deviations between the synchronized frequency and the individual frequencies are ε-small, when the deviations from the average oscillator frequency $\langle\omega\rangle = N^{-1}\sum_i \omega_i$ are of the order ε as assumed in the previous derivations:

$$\varepsilon\Delta\omega_j = \omega_j - \Omega = \frac{\sum_i u_{1i}(\omega_j - \omega_i - \varepsilon\Gamma_i(\boldsymbol{0}))}{\sum_i u_{1i}} = \varepsilon\frac{\sum_i u_{1i}(\Delta\langle\omega\rangle_j - \Delta\langle\omega\rangle_i - \Gamma_i(\boldsymbol{0}))}{\sum_i u_{1i}}. \tag{23}$$

This shows that the averaging in eq. 13 is justified. In a similar way we obtain:

$$\Delta\widetilde{\omega}_j = \omega_j\varepsilon^{-1} + \Gamma_j(\boldsymbol{0}) - \Omega\varepsilon^{-1} = \frac{\sum_i u_{1i}\big(\Delta\langle\omega\rangle_j + \Delta\langle\Gamma(\boldsymbol{0})\rangle_j - \Delta\langle\omega\rangle_i - \Delta\langle\Gamma(\boldsymbol{0})\rangle_i\big)}{\sum_i u_{1i}}$$

$$= \Delta\langle\omega\rangle_j + \Delta\langle\Gamma(\boldsymbol{0})\rangle_j - \frac{\sum_i u_{1i}(\Delta\langle\omega\rangle_i + \Delta\langle\Gamma(\boldsymbol{0})\rangle_i)}{\sum_i u_{1i}}, \tag{24}$$

where $\Delta\langle\omega\rangle_j = \varepsilon^{-1}(\langle\omega\rangle - \omega_j)$, $\Delta\langle\Gamma(\boldsymbol{0})\rangle_j = \langle\Gamma(\boldsymbol{0})\rangle - \Gamma_j(\boldsymbol{0})$ and $\langle\Gamma(\boldsymbol{0})\rangle = N^{-1}\sum_i \Gamma_i(\boldsymbol{0})$. Thus the vector $\boldsymbol{\Delta\widetilde{\omega}}$ is mainly determined by the deviations from the average values of the oscillator frequency and the coupling contribution.

Eq. 19 can be simplified by noting that $\boldsymbol{M}^{+}\boldsymbol{M} = \boldsymbol{I} - \boldsymbol{v}_1\boldsymbol{v}_1^T$:

$$\boldsymbol{\psi}^0 = -\boldsymbol{M}^{+}\boldsymbol{\Delta\widetilde{\omega}} + \boldsymbol{v}_1^T\boldsymbol{v}_1\boldsymbol{w} = -\boldsymbol{M}^{+}\boldsymbol{\Delta\widetilde{\omega}} + (\langle\boldsymbol{w}\rangle, \ldots, \langle\boldsymbol{w}\rangle)^T. \tag{25}$$

Therefore, as expected the solution is not unique with respect to a simultaneous shift of all oscillators. For convenience we consider the solution with $\boldsymbol{w} = \boldsymbol{0}$.

To characterize the distribution some more we now assume that $\boldsymbol{\Delta\widetilde{\omega}}$ is Gaussian distributed with the simple variance $\sigma_{\Delta\widetilde{\omega}}^2\boldsymbol{I}$. Since $\boldsymbol{\psi}^0$ is a linear transformation of $\boldsymbol{\Delta\widetilde{\omega}}$ its distribution is also Gaussian with variance:

$$\boldsymbol{M}^{+}\sigma_{\Delta\widetilde{\omega}}\boldsymbol{I}(\boldsymbol{M}^{+})^T = \sigma_{\Delta\widetilde{\omega}}\boldsymbol{V}(\boldsymbol{\Sigma}^{+})^2\boldsymbol{V}^T = \sigma_{\Delta\widetilde{\omega}}\sum_{i=2}^{N}\frac{1}{\sigma_i}\boldsymbol{v}_i^T\boldsymbol{v}_i. \tag{26}$$

This shows that the variance is mostly determined by the squared singular values that are near zero. Moreover, the singular vectors corresponding to these near-zero eigenvalues are near to the kernel vector $\boldsymbol{v}_1 = 1/\sqrt{N}\,(1, \ldots, 1)^T$. This can be seen from the definition of the singular vectors:

$$\boldsymbol{M}\boldsymbol{v}_i = \sqrt{\sigma_i}\boldsymbol{u}_i, \|\boldsymbol{u}_i\| = \|\boldsymbol{v}_i\| = 1,\ i = 2, \ldots, N. \tag{27}$$

For very small singular values the equation is similar to the equation determining the kernel vector. This implies that $\boldsymbol{v}_i^T\boldsymbol{v}_i$ will be very similar to $\boldsymbol{v}_1^T\boldsymbol{v}_1 = 1/N\,\boldsymbol{1}$ (where $\boldsymbol{1}$ is a matrix filled with ones). Thus, the near-zero singular values dominate the overall variance of the stable phase distribution $\boldsymbol{\psi}_0$.

In the case of a symmetric matrix $\boldsymbol{M}$, which arises for a symmetric bidirectional coupling with all couplings similar, the singular values and their vectors can be replaced by the eigenvalues and eigenvectors of $\boldsymbol{M}$. Numerical calculations show this analytically derived behavior [6].

4. Special cases of coupling

Next, we consider some special but common cases of coupling. If all oscillators are coupled symmetrically, additively and in the same way, the synchronized frequency in eq. 22 is:

$$\Omega = \langle\omega\rangle + \varepsilon\langle k\rangle\Gamma(0). \tag{28}$$

The system in eq. 16 simplifies to:

$$\boldsymbol{\Delta\widetilde{\omega}} = (\Delta\langle\omega\rangle_1 + \Delta\langle k\rangle_1\Gamma(0), \ldots, \Delta\langle\omega\rangle_N + \Delta\langle k\rangle_N\Gamma(0))^T, \tag{29}$$

$$\boldsymbol{M} = \kappa\boldsymbol{L}, \tag{30}$$

where, $\Gamma(0) = \frac{1}{2\pi}\int_0^{2\pi}\boldsymbol{Q}(t)\boldsymbol{C}(\boldsymbol{x}^0(t))\,dt$, $\kappa = \frac{1}{2\pi}\int_0^{2\pi}\boldsymbol{Q}(t)\frac{\partial\boldsymbol{C}}{\partial\boldsymbol{x}}(\boldsymbol{x}^0(t))\boldsymbol{F}(\boldsymbol{x}^0(t))\,dt$, $\Delta\langle k\rangle_i$ is the deviation of the i-th oscillator from the average number of connected oscillators $\langle k\rangle$ and

$L_{ij} = A_{ij} - (\sum_j A_{ij})\delta_{ij}$ are the entries of the symmetric Laplacian matrix $\boldsymbol{L}$ of the oscillator network defined by the adjacency matrix $\boldsymbol{A}$. Thus, in this case the matrix $\boldsymbol{M}$ is identical to the Laplacian $\boldsymbol{L}$ of the oscillator network up to a constant factor and the eigenvalues of $\boldsymbol{L}$ determine the dynamics of synchronization. Therefore, it suffices to analyze the Laplacian of the network in this case.

In our model the bidirectional couplings between the oscillators are weighted by the number of connections k_i the i-th oscillator has and therefore are not symmetric (see manuscript, Section: Oscillator Network, eq. 3). However, since we can write the matrix $\boldsymbol{M}$ as follows:

$$\boldsymbol{M} = \kappa\, \mathrm{diag}(k_1{}^{-1}, \dots, k_N{}^{-1})\boldsymbol{L} = \kappa \boldsymbol{K}^{-1}\boldsymbol{L}, \tag{31}$$

the kernel vector $\boldsymbol{u}_1$ of $\boldsymbol{M}^T = \kappa \boldsymbol{L}\boldsymbol{K}^{-1}$ is simply given by $(k_1, \dots, k_N)$. Thus the synchronized frequency from eq. 22 is given by:

$$\Omega = \frac{\sum_i k_i \omega_i}{\sum_i k_i} + \varepsilon\Gamma(0).$$

The weighting leads to the following linear system:

$$\dot{\boldsymbol{\psi}} = \boldsymbol{\Delta\tilde{\omega}} + \kappa \boldsymbol{K}^{-1}\boldsymbol{L}\boldsymbol{\psi}, \tag{32}$$

$$\boldsymbol{\Delta\tilde{\omega}} = (\Delta\langle\omega\rangle_1 - \frac{\sum_i k_i \Delta\langle\omega\rangle_i}{\sum_i k_i}, \dots, \Delta\langle\omega\rangle_N - \frac{\sum_i k_i \Delta\langle\omega\rangle_i}{\sum_i k_i})^T. \tag{33}$$

For our model the system simplifies further due to the linear coupling between the oscillators and the simple one-to-one coupling of oscillator dimensions:

$$\frac{\partial \boldsymbol{C}}{\partial x} = \begin{pmatrix} 1 & 0 \\ 0 & 1 \end{pmatrix}, \kappa = \frac{1}{2\pi}\int_0^{2\pi} \boldsymbol{Q}(t) \cdot \boldsymbol{F}(\boldsymbol{x}(t))\, dt = 1. \tag{34}$$

Here the last identity follows from the normalization condition $\boldsymbol{Q}(t) \cdot \boldsymbol{F}(\boldsymbol{x}(t)) = 1$. Therefore, in this case the matrix $\boldsymbol{M}$ is identical to $\boldsymbol{L}$. Moreover, for our model it is easy to show that:

$$\Gamma(0) = \frac{1}{2\pi}\int_0^{2\pi} \boldsymbol{Q}(t)\boldsymbol{C}(\boldsymbol{x}^0(t))\, dt$$

$$= \frac{1}{2\mu}\int_0^{2\pi} -\frac{1}{r^0}\begin{pmatrix} \sin(\varphi^0(t)) \\ \cos(\varphi^0(t)) \end{pmatrix} r^0 \begin{pmatrix} \cos(\varphi^0(t)) & 0 \\ 0 & \sin(\varphi^0(t)) \end{pmatrix} dt = 0. \tag{35}$$

5. Spectral Graph Analysis and Synchronization

The community structure of undirected networks can be characterized by the eigenvalue spectrum of the network's symmetric Laplacian matrix $\boldsymbol{L}$ (see Eq. 5 of main text). These eigenvalues can be ordered by increasing magnitude and due to the special construction of the

Laplacian it always has a trivial zero eigenvalue. Any further zero eigenvalue indicates that the graph is partitioned into disconnected components. However, also eigenvalues that are near-zero indicate components that are loosely connected. These eigenvalues can be related to the so called algebraic connectivity of the network [9]. Especially, the second smallest eigenvalue bounds the isoperimetric number of the graph via the cheeger inequalitiy [10]. In general, a small isoperimetric number indicates 'bottlenecks' in the graph. The network can be subdivided into communities via these bottlenecks. This is done by considering the eigenvectors associated with the eigenvalues, also known as Fiedler vectors. Nodes can be assigned to specific communities according to the values of the eigenvectors and in fact there are algorithms that partition graphs according to the eigenvector and eigenvalue values [11, 12].

For non-symmetric networks, as in our study due to the local mean-field coupling, the singular value spectrum becomes important. We have calculated the singular value spectra for both the summer and winter topology for 25 replicates of our network structure (Section 7, Figure S6). The large fraction of singular values near 1 corresponds to groups of oscillators that are well connected to each other. In our case the local mean field coupling leads to a normalization of the singular values around 1 as was discussed in a previous study [7]. It should be noted that a fully synchronized network still possesses one trivial zero-eigenvalue/singularvalue, which describes a simultaneous phase advance in all oscillators. The winter topology shows only the trivial zero-eigenvalues (25, one for each replicate) at zero (Section 7, Figure S6B). On the other hand for the summer topology one observes additional near-zero singular values, leading to a larger bin of the histogram around zero (>25, Section 7, Figure S6A). With our theoretical results from the previous sections 2-4 it is now clear that for the summer topology the phase distribution is broader, since small singular values lead to a large variance in the phase distribution (see Eq. 26).

It was shown previously that the spectral properties of the Laplacian also affect the synchronization dynamics [6, 7]. In particular, the individual eigenvalues can be related to separate timescales of synchronization of oscillator communities associated to the Fiedler vectors. In these numerical studies, especially graphs with community structure, indicated by small non-zero eigenvalues as in the summer-topology (Section 7, Figure S6A), showed worse synchronization behavior in the sense that stronger coupling is needed to achieve a similar amount of phase synchronization as in a network with no community structure. This is intuitively to be expected since the bottlenecks limit the coupling from one community to the other and in general the communities are connected only via a few nodes. These bottlenecks also intuitively explain why the phase distribution is broader in the summer topology. Moreover, for the winter topology a gap between the zero and non-zero singular values can be observed (Section 7, Figure S6B), indicating fast synchronization of the SCN cells in winter, since slow timescales are missing.

6. Entrainment of a single amplitude-phase oscillator

In this section, we want to analyze the entrainment behavior of a single oscillator of the type considered in our study (see Eqs. (1) and (2) in the main text). Ultimately, we aim at deriving analytical expressions for the entrainment border of the entrainment region (commonly referred to as Arnold tongue). In line with our study, we consider entrainment by square-shaped signals. The amplitude-phase oscillators we consider are generally given by:

$$\dot{r} = \lambda f(r) \tag{36}$$

$$\dot{\varphi} = \gamma(\cos\varphi/2)^2 + 2\pi/T. \tag{37}$$

Here T determines the offset such that the oscillator has a period of τ or frequency $\omega = 2\pi/\tau$. Circadian rhythms always have $\omega < 1$. Integrating the system by separation of variables and relating the period τ to 2π we derive $T = \pi/\omega^2(\gamma + \sqrt{4\omega^2 + \gamma^2})$. For the oscillator used in our study $f(r) = r(A - r)$, which is commonly referred to as the Poincaré oscillator. Applying a forcing $bg(t)$, where b is the entrainment amplitude and $g(t)$ a function with frequency $\Omega = \frac{2\pi}{\tau_\mathrm{e}}$, to the x-coordinate (rectangular coordinates) the system in polar coordinates is given by:

$$\dot{r} = \lambda f(r) + \cos\varphi\, bg(t) \tag{38}$$

$$\dot{\varphi} = \frac{\gamma}{2} + 2\pi/T + \frac{\gamma}{2}\cos\varphi - \frac{1}{r}\sin\varphi\, bg(t) = \alpha + \beta\cos\varphi - \frac{1}{r}\sin\varphi\, bg(t). \tag{39}$$

The key to analyzing this system is to separate slow time-scales of amplitude and/or phase adaptation and fast time-scales of external forcing. The method of choice is the averaging technique introduced by Krylov and Bogolyubov [13]. However, a straightforward application of this method is hindered by the fast terms involving γ. A solution to this problem is to project the phase of the forced system onto a suitable chosen phase of the unperturbed limit cycle. A suitable chosen phase is here defined as a constantly (with time) increasing variable [1].

Such a projection can be found by considering the unperturbed phase in Eq. (37). Performing the first steps of separation of variables we obtain:

$$F(\varphi) = \int_0^{\varphi} \frac{1}{\alpha + \beta\cos\varphi'}\, d\varphi' = t. \tag{40}$$

This shows that a suitable phase is given by t with the corresponding projection $F(\varphi)$. We now consider the projection $F(\varphi(t)) = \tilde{t}$ of a solution of Eqs. (38) and (39) and compute its time derivative:

$$\frac{d\tilde{t}}{dt} = \left.\frac{dF(\varphi)}{d\varphi}\right|_{\varphi(t)} \dot{\varphi} = 1 - \frac{1}{\alpha + \beta\cos\varphi(t)}\frac{1}{r}\sin\varphi(t)\, bg(t). \tag{41}$$

The term $-\frac{1}{r}\frac{\sin\varphi(t)}{\alpha+\beta\cos\varphi(t)} = -\frac{1}{r}\frac{\sin F^{-1}(\tilde{t})}{\alpha+\beta\cos F^{-1}(\tilde{t})} = \mathrm{iPRC}_x(\tilde{t})$ is the infinitesimal phase response curve of the oscillator with respect to a perturbation in the *x*-coordinate, already introduced in section 1 Eq. (3). Performing the integration in Eq. (40) we obtain:

$$F^{-1}(\tilde{t}) = 2\tan^{-1}\left(\frac{\alpha+\beta}{\sqrt{\alpha^2-\beta^2}}\tan\left(1/2\sqrt{\alpha^2-\beta^2}\tilde{t}\right)\right) = 2\tan^{-1}\left(\frac{\alpha+\beta}{\omega}\tan(\omega/2\tilde{t})\right) \quad (42)$$

and after some tedious algebra we derive a simple expression for the phase response:

$$\mathrm{iPRC}_x(\tilde{t}) = -\frac{1}{\omega r}\sin\omega\tilde{t}. \quad (43)$$

For completeness, we also give the phase response in the *y*-coordinate, which can be obtained in a similar manner:

$$\mathrm{iPRC}_y(\tilde{t}) = \frac{1}{\omega^2 r}(-\beta+\alpha\cos\omega\tilde{t}). \quad (44)$$

This shows that in general a forcing in the *y*-coordinate is not preferable since it shifts the entrainment region away from the intrinsic period towards lower periods.

After defining the new phase as $\tilde{\varphi} = \omega\tilde{t}$, the system in Eqs. (38) and (39) is rewritten as:

$$\dot{r} = \lambda f(r) + \cos F^{-1}(\tilde{\varphi})\, bg(t) = \lambda f(r) + \frac{-\gamma+\sqrt{\gamma^2+4\omega^2}\cos\tilde{\varphi}}{\sqrt{\gamma^2+4\omega^2}-\gamma\cos\tilde{\varphi}} bg(t) \quad (45)$$

$$\dot{\tilde{\varphi}} = \omega - \frac{1}{r}\sin\tilde{\varphi}\, bg(t). \quad (46)$$

It now makes sense to introduce the phase difference to the external forcing $\psi = \tilde{\varphi} - \Omega t$ and consider several limit cases to obtain analytical expressions for the entrainment range.

First of all, let us consider rigid oscillators ($\lambda \gg 1$). Then the amplitude of the oscillator will not change under forcing and will be $f(r^0) = 0 \Leftrightarrow r^0 = A$ ($r^0 = 0$ is an unstable solution). Thus, the above system reduces to one equation for the phase difference:

$$\dot{\psi} = \omega - \Omega - \frac{1}{A}\sin(\Omega t + \psi)\, bg(t) = \Delta - \frac{1}{A}\sin(\Omega t + \psi)\, bg(t). \quad (47)$$

If b and Δ are small then ψ will be a slow moving variable compared to the forcing and thus we can average over one external period τ_e:

$$\dot{\psi} = \Delta - \frac{b}{A}\frac{1}{\tau_\mathrm{e}}\int_0^{\tau_\mathrm{e}}\sin(\Omega t + \psi)\, g(t)dt. \quad (48)$$

Here ψ has to be seen as the variable on the slow time scale. The integral is performed easily for a sinusoidal or square-shaped forcing signal. To be specific we here consider a square-shaped function $g(t)$ with period τ_e that starts with 1 at $t = 0$ and is set to 0 for $t = t_p$. Here t_p is the width of the entraining signal (see also main text). Then the integral is given by:

$$\frac{1}{\tau_\mathrm{e}}\int_0^{\tau_\mathrm{e}}\sin(\Omega t + \psi)\, g(t)dt = \frac{\sin(t_p/\tau_\mathrm{e}\pi)}{\pi}\cos\left(\psi + \frac{t_p}{\tau_\mathrm{e}}\pi - \frac{\pi}{2}\right) = \frac{\sin(w\pi)}{\pi}\sin(\psi + w\pi). \quad (49)$$

Here we defined $w = t_p/\tau_\mathrm{e}$. This implies that the entrainment region for rigid oscillators under rectangular forcing is given by:

$$b = \frac{\pi}{\sin(w\pi)} A|\Delta|. \tag{50}$$

Let us now consider weak oscillators ($\lambda \ll 1$). In this case, the amplitude dynamics in Eq. (45) cannot be neglected. However, if b and λ are small the amplitude dynamics can be treated by the same averaging method we already used for the phase difference dynamics:

$$\dot{r} = \lambda f(r) + b\frac{1}{\tau_\mathrm{e}}\int_0^{\tau_\mathrm{e}} \frac{-\gamma+\sqrt{\gamma^2+4\omega^2}\cos(\Omega t+\psi)}{\sqrt{\gamma^2+4\omega^2}-\gamma\cos(\Omega t+\psi)} g(t)dt, \tag{51}$$

$$\dot{\psi} = \Delta - \frac{b}{r}\frac{\sin(w\pi)}{\pi}\sin(\psi + w\pi). \tag{52}$$

Again r and ψ should be interpreted as variables on the slow time scale. Even for a rectangular forcing $g(t)$ the integral is rather complicated, although it can be solved exactly. Therefore, we consider two additional limit cases: sinusoidal oscillators with $\gamma = 0$ and spiking oscillators with $\gamma \gg 1$. For $\gamma = 0$ the system reduces to the original system in Eqs. (38) and (39) and the integral under the previously defined rectangular forcing is given by:

$$\frac{1}{\tau_\mathrm{e}}\int_0^{\tau_\mathrm{e}} \cos(\Omega t + \psi)\, g(t)dt = \frac{\sin(w\pi)}{\pi}\cos(\psi + w\pi). \tag{53}$$

Thus, we obtain the following equations for the steady state solution:

$$-\lambda f(r) = b\frac{\sin(w\pi)}{\pi}\cos(\psi + w\pi) = \tilde{b}\cos(\tilde{\psi}), \tag{54}$$

$$\Delta r = \tilde{b}\sin(\tilde{\psi}). \tag{55}$$

We have:

$$\tan(\tilde{\psi}) = \frac{\sin(\tilde{\psi})}{\cos(\tilde{\psi})} = \frac{-\Delta \mathrm{r}}{\lambda f(r)} \Leftrightarrow \tilde{\psi} = \tan^{-1}\left(\frac{-\Delta \mathrm{r}}{\lambda f(r)}\right), \tag{56}$$

$$\Delta r\frac{1}{\tilde{b}} = \sin\left(\tan^{-1}\left(\frac{\Delta r}{\lambda f(r)}\right)\right) = \frac{-\Delta r}{\sqrt{\left(\lambda f(r)\right)^2+(\Delta \mathrm{r})^2}} \Leftrightarrow \tilde{b}^2 = \left(\lambda f(r)\right)^2 + (\Delta \mathrm{r})^2 = h(r). \tag{57}$$

For the Poincaré oscillator in our study we have: $h(r) = r^2(\Delta^2 + \lambda^2(A - r)^2)$. Obviously this function is always larger or equal to 0, goes through the origin (0,0) and has a maximum and a minimum between $A/2$ and A at: $\frac{3}{4}A - \sqrt{(1/4A)^2 - 1/2(\Delta/\lambda)^2}$ and $\frac{3}{4}A + \sqrt{(1/4A)^2 - 1/2(\Delta/\lambda)^2}$, respectively. These minima and maxima exist only for $|\Delta| < \sqrt{2}/4\mathrm{A}\lambda$. Thus, Eq. (57) that determines the entrained amplitude has three solutions for $|\Delta| < \sqrt{2}/4\mathrm{A}\lambda$ and one solution for $|\Delta| \geq \sqrt{2}/4\mathrm{A}\lambda$. Therefore, the entrainment region is determined by the linear stability of these solutions. The coefficients of the quadratic characteristic polynomial of the system are given by:

$$a_1 = \frac{1}{r}\tilde{b}\cos\tilde{\psi} - f'(r) = -\frac{\lambda f(r)}{r} - f'(r) = \lambda(3r - 2A), \tag{58}$$

$$a_2 = \left(\frac{\tilde{b}}{r}\sin\tilde{\psi}\right)^2 - f'(r)\frac{\tilde{b}}{r}\cos\tilde{\psi} = \Delta^2 + \frac{f'(r)f(r)}{r} = 2\lambda^2 r^2 - 3A\lambda^2 r + A^2\lambda^2 + \Delta^2. \tag{59}$$

The first coefficient is negative for $r < 2/3A$. According to Hurwitz' criterion solutions with $r < 2/3A$ will be unstable. The second coefficient is negative between the maxima and minima of the function $h(r)$ in Eq. (57): $\frac{3}{4}A - \sqrt{(1/4A)^2 - 1/2(\Delta/\lambda)^2} < r < \frac{3}{4}A + \sqrt{(1/4A)^2 - 1/2(\Delta/\lambda)^2}$. Therefore, according to Hurwitz' criterion these solutions will be saddle points and unstable. Thus, for $|\Delta| \geq \sqrt{2}/4A\lambda$ the entrainment border is given by $r = 2/3A$. For $|\Delta| < \sqrt{2}/4A\lambda$ the situation is more complex since additionally to the stable solution right to the minimum of the function $h(r)$ in Eq. (57), there can be an additional stable solution if $\frac{3}{4}A - \sqrt{(1/4A)^2 - 1/2(\Delta/\lambda)^2} > 2/3A$. This is the case for $1/3A\lambda < |\Delta| \leq \sqrt{2}/4A\lambda$. We find that for $1/3\sqrt{1/2(11\sqrt{33} - 61)}A\lambda \leq |\Delta| \leq \sqrt{2}/4A\lambda$. the function value $h(2/3A)$ is smaller than $h(\frac{3}{4}A + \sqrt{(1/4A)^2 - 1/2(\Delta/\lambda)^2})$ and therefore the first function value (at $h(2/3A)$) corresponds to the larger entrainment border. However, since $1/3\sqrt{1/2(11\sqrt{33} - 61)} \approx 0.349$ is very near to $\sqrt{2}/4 \approx 0.354$ we may also use the simpler bound $\sqrt{2}/4A\lambda$ for small $A\lambda$. Summarizing the discussion, the entrainment borders for weak sinusoidal Poincaré oscillators under rectangular forcing are given by:

$$b = \frac{\pi}{\sin(w\pi)} r_0\sqrt{\Delta^2 + \lambda^2(A - r_0)^2}, r_0 = \frac{3}{4}A + \sqrt{\left(\frac{1}{4}A\right)^2 - \frac{1}{2}\left(\frac{\Delta}{\lambda}\right)^2} \quad \Leftrightarrow |\Delta| < \sqrt{2}/4A\lambda \tag{60}$$

$$b = \frac{\pi}{\sin(w\pi)} r_0\sqrt{\Delta^2 + \lambda^2(A - r_0)^2}, r_0 = \frac{2}{3}A \quad \Leftrightarrow |\Delta| \geq \sqrt{2}/4A\lambda. \tag{61}$$

We can see that since $r_0 \leq A$ the entrainment region of weak sinusoidal Poincaré oscillators is always larger than that of the corresponding rigid oscillators. The reason for this is the decrease of the entrained amplitude near the entrainment border.

Let us now consider weak spiking oscillators with $\gamma \gg 1$. We then have to deal with the full integral in Eq. (51). The integral can be calculated as:

$$\Gamma(\psi) = -\mathrm{w}\frac{\sqrt{\gamma^2+4\omega^2}}{\gamma} + \frac{2\omega}{\pi\gamma}\left(\cot^{-1}\left(\frac{2\omega\cot\left(\frac{\psi}{2}+w\pi\right)}{\gamma+\sqrt{\gamma^2+4\omega^2}}\right) - \cot^{-1}\left(\frac{2\omega\cot\left(\frac{\psi}{2}\right)}{\gamma+\sqrt{\gamma^2+4\omega^2}}\right)\right). \tag{62}$$

The terms involving the cotangents can be bounded by 0 and π. Thus, we have $-\mathrm{w}\frac{\sqrt{\gamma^2+4\omega^2}}{\gamma} \leq \Gamma(\psi) \leq= -\mathrm{w}\frac{\sqrt{\gamma^2+4\omega^2}}{\gamma} + \frac{2\omega}{\gamma}$ and for $\gamma \gg 1$ a good approximation is $\Gamma(\psi) \cong -\mathrm{w}$. Using this approximation in Eq. (51) we have the following equations determining the steady-state solutions of the slow variables:

$$\lambda f(r) = bw, \tag{63}$$

$$\Delta = \frac{\tilde{b}}{r}\sin(\tilde{\psi}). \tag{64}$$

Here, the equations decouple and the steady-state solutions can be easily found. For the Poincaré oscillator amplitude, we obtain a stable (+) and an unstable (-) solution at:

$$r_0 = \frac{A}{2} \pm \sqrt{\left(\frac{A}{2}\right)^2 - b\frac{w}{\lambda}} \tag{65}$$

Here an interesting phenomenon appears for a weak spiking oscillator forced by square-shaped signals. For all initial amplitudes below the unstable solution, $r \xrightarrow{t\to\infty} 0$ on the slow time scale. Moreover, at:

$$b = \left(\frac{A}{2}\right)^2 \frac{\lambda}{w} \tag{66}$$

the stable solution vanishes and for all initial conditions on the slow time scale $r \xrightarrow{t\to\infty} 0$. Let us first consider the stable solution for $b \leq \left(\frac{A}{2}\right)^2 \frac{\lambda}{w}$. Then the entrainment border is given by solving Eqs. (63) and (64) with the sine term set to 1:

$$b = \frac{\pi}{\sin(w\pi)} A|\Delta| - \frac{\mathrm{w}}{\lambda}\left(\frac{\pi}{\sin(w\pi)}\Delta\right)^2, |\Delta| \leq \frac{\sin(w\pi)}{\pi}\frac{A}{2}\frac{\lambda}{w}. \tag{67}$$

Notice that as in the case of a sinusoidal oscillator, weak spiking oscillators show a larger entrainment region. Now let us consider the case where $r \xrightarrow{t\to\infty} 0$, either because the initial conditions are below the unstable steady state or because the forcing strength is larger than the bound in Eq. (66). This case can be interpreted as the forcing being stronger than the internal dynamics of the oscillator. In this case, as $r \to 0$ the averaging technique breaks down since ψ in Eq. (52) will no longer be a slow moving variable due to the b/r term. Thus, we have to consider the full system in Eqs. (45) and (46). However, since $r \ll 1$ and $\lambda \ll 1$ we can neglect the $f(r)$ term:

$$\dot{r} = \frac{\sqrt{\gamma^2+4\omega^2}\cos\tilde{\varphi} - \gamma}{\sqrt{\gamma^2+4\omega^2} - \gamma\cos\tilde{\varphi}} bg(t) \tag{68}$$

$$\dot{\tilde{\varphi}} = \omega - \frac{1}{r}\sin\tilde{\varphi}\, bg(t). \tag{69}$$

For $\gamma \gg 1$ an attractor of this system always exists. Obviously, during $g(t) = 0$ the amplitude does not change, while the phase moves according to its internal frequency ω. Now, consider the onset of the external forcing ($g(\tau_{\mathrm{e}}) = 1$). Assume that at this point $\tilde{\varphi}$ is somewhere near π. Then $\dot{r} \approx -1b = -b$ and thus $r \to 0$. This implies that the phase dynamics in Eq. (69) becomes very fast, i.e. a phase-resetting occurs. It now depends on how near $\tilde{\varphi}$ is to π, whether a) $\dot{\tilde{\varphi}} < 0$ and thus a resetting to the old phase occurs, or b) $\dot{\tilde{\varphi}} > 0$ and thus a resetting to a new phase with $\tilde{\varphi} > 2\pi$ occurs. After this very fast resetting and as long as $g(t) = 1$ ($t \leq t_p$) the dynamics will stay at the steady state determined by setting Eqs. (68) and (69) to zero:

$$\dot{r} = 0 \Leftrightarrow \frac{\sqrt{\gamma^2+4\omega^2}\cos\tilde{\varphi}-\gamma}{\sqrt{\gamma^2+4\omega^2}-\gamma\cos\tilde{\varphi}} = 0 \Leftrightarrow \tilde{\varphi}_0 = \pm\cos^{-1}\left(\frac{\gamma}{\sqrt{\gamma^2+4\omega^2}}\right), \tag{70}$$

$$\dot{\tilde{\varphi}} = 0 \Leftrightarrow r_0 = \frac{b}{\omega}\sin\tilde{\varphi}_0 \Leftrightarrow r_0 = 2b\sqrt{\frac{1}{\gamma^2+4\omega^2}}. \tag{71}$$

Here only the positive phase is a stable solution. We now have characterized the behavior of this low amplitude attractor. Next, we approximately derive the entrainment bounds. As we already have seen above in case a) the phase will always be resetted to $\tilde{\varphi}_0$ and thus $|\psi(t)| = |\tilde{\varphi}(t) - \Omega t| \xrightarrow{t\to\infty} \infty$. In a strict sense, this means that no entrainment is possible, since the oscillator phase cannot follow the external phase due to resetting. On the other hand consider case b) in this case at the k-th resetting the phase will be shifted to a point above 2π to the steady state at $\tilde{\varphi}_0 + (k+1)2\pi$. This implies $|\psi(t)| = |\tilde{\varphi}(t) - \Omega t| < 2\pi$ and thus entrainment of the oscillator. Therefore, the upper condition for the entrainment (in a strict sense) is approximately given by:

$$\tilde{\varphi}(\tau_\mathrm{e}) = \tilde{\varphi}_0 + \omega\left(\tau_\mathrm{e} - t_p\right) > \pi - \tilde{\varphi}_0 \Leftrightarrow \Omega < \omega(1-w)\frac{2\pi}{\pi - 2\tilde{\varphi}_0}. \tag{72}$$

On the other hand, since we only consider 1:1 entrainment, a lower bound can be given as:

$$\tilde{\varphi}(\tau_\mathrm{e}) = \tilde{\varphi}_0 + \omega\left(\tau_\mathrm{e} - t_p\right) < 3\pi - \tilde{\varphi}_0 \Leftrightarrow \Omega > \omega(1-w)\frac{2\pi}{3\pi - 2\tilde{\varphi}_0}. \tag{73}$$

These bounds becomes more exact the larger γ. However, as mentioned above the bound in Eq. (72) is for entrainment seen in a strict sense. This means that the phase $\tilde{\varphi}(t)$ of the oscillator, measured as the phase of the unperturbed oscillator, follows the external phase. To overcome this limitation we may look at each resetting event as a resetting of $\tilde{\varphi}(t)$ to $\tilde{\varphi}_0$ during which $\tilde{\varphi}(t)$ crosses 2π. Then the bound in Eq. (72) is meaningless, which means that entrainment to periods smaller than the internal period is always possible for weak, spiking oscillators due to the domination of the dynamics by the rectangular forcing.

All entrainment borders of the individual oscillator in the specific cases considered are shown in Figure S7 (Section 7).

7. Supplementary Figures S1-S11

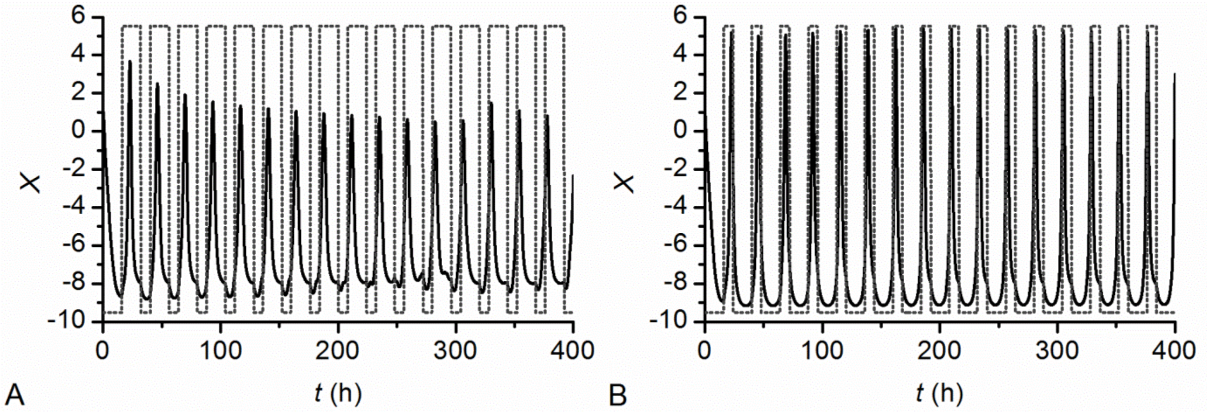

Figure S1. Transient time evolution of the mean-field for summer – δ=0.005, t_p =16 (A) and winter – δ=0.01, t_p =8 (B) conditions. All simulations were started with initial conditions randomly distributed around x = 1 and y = 0, according to a normal distribution with a standard deviation of 0.2. Just a few periods are necessary for the system to get into its final dynamical state.

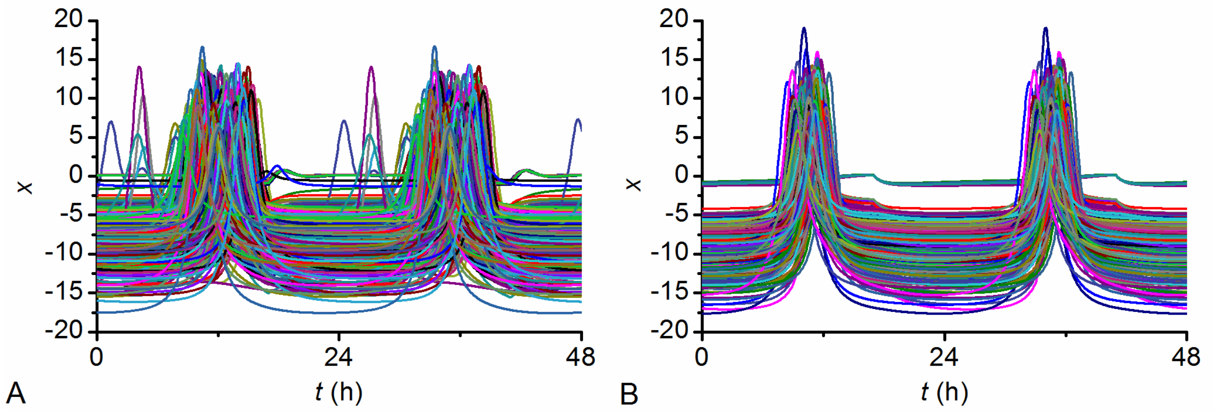

Figure S2. Oscillatory activity patterns of all N = 600 SCN neurons. (A) Summer conditions: $t_p = 16$, $\delta = 0.005$ and (B) winter conditions: $t_p = 8$, $\delta = 0.01$.

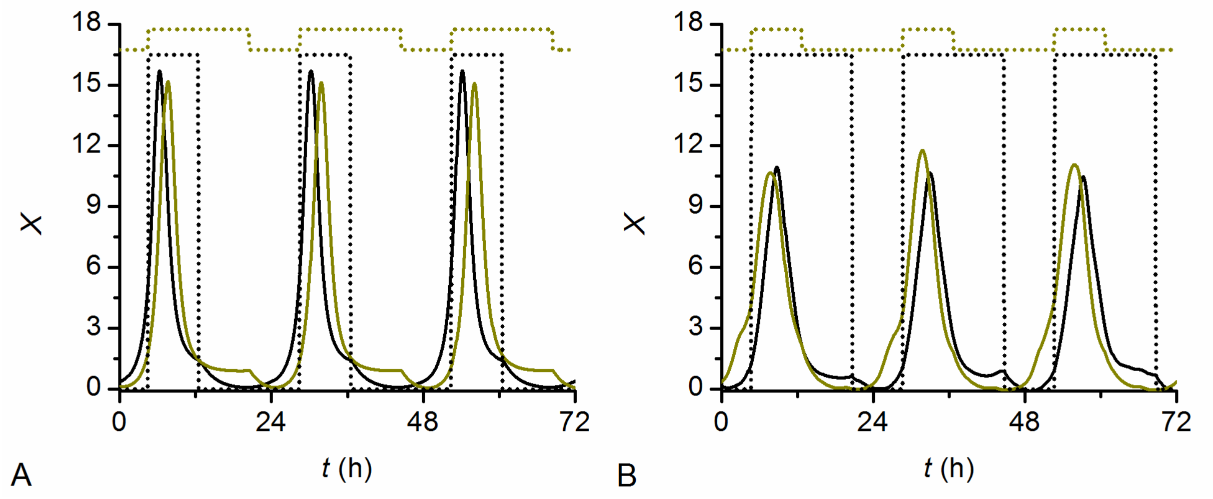

Figure S3. Global activity patterns of the SCN. Time courses of the mean field for winter– $\delta = 0.01$ (A) and summer – $\delta = 0.005$ conditions (B). In both cases different durations of the light signal have been applied ($t_p = 8$ and $t_p = 16$). Evidently, the network structure is the crucial factor defining the shape of the global activity, whereas the duration of the light input has an almost negligible effect.

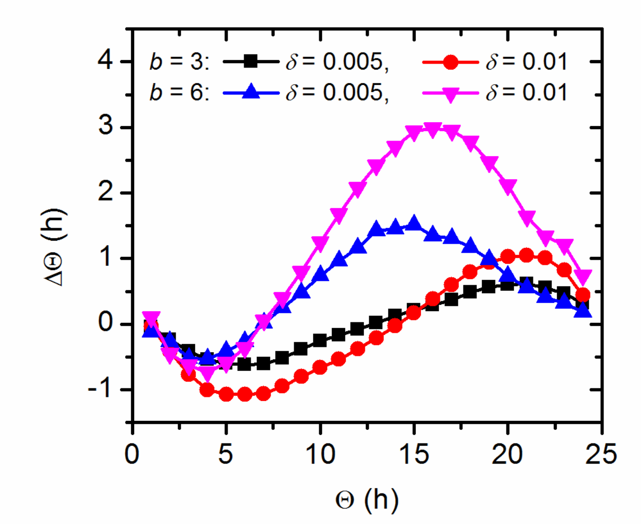

Figure S4. Phase response curves of the SCN network obtained at different pulse amplitudes. The curves indicate changes in the phase of the mean field signal for summer ($\delta = 0.005$) and winter ($\delta = 0.01$) conditions. In both cases the pulse had a duration of 4 h.

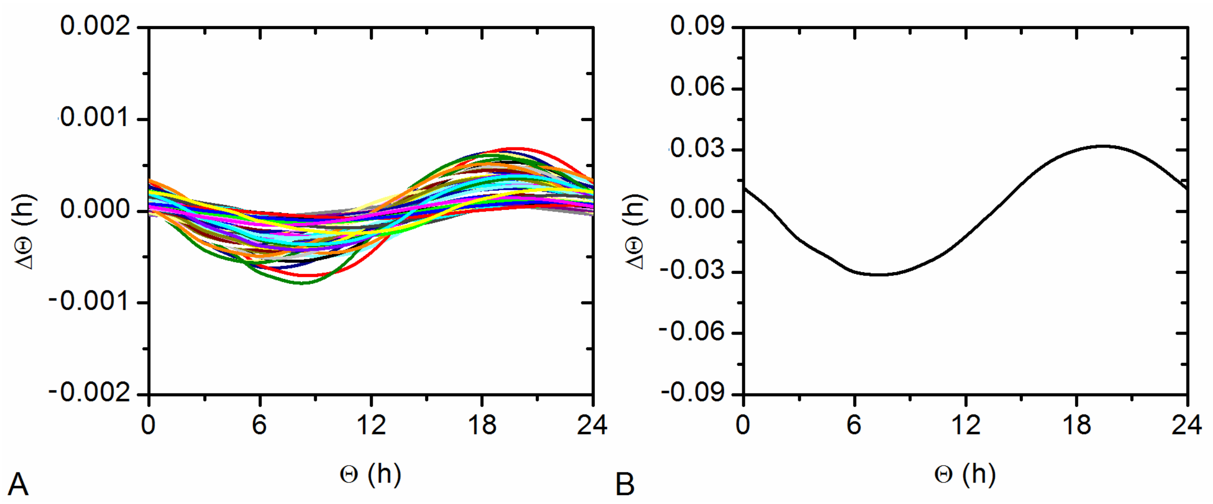

Figure S5. Instantaneous phase response curves of all light-receiving neurons. Individual PRCs for $\delta = 0.0035$ (A) and the corresponding summed up phase response (B). Time is scaled to circadian time.

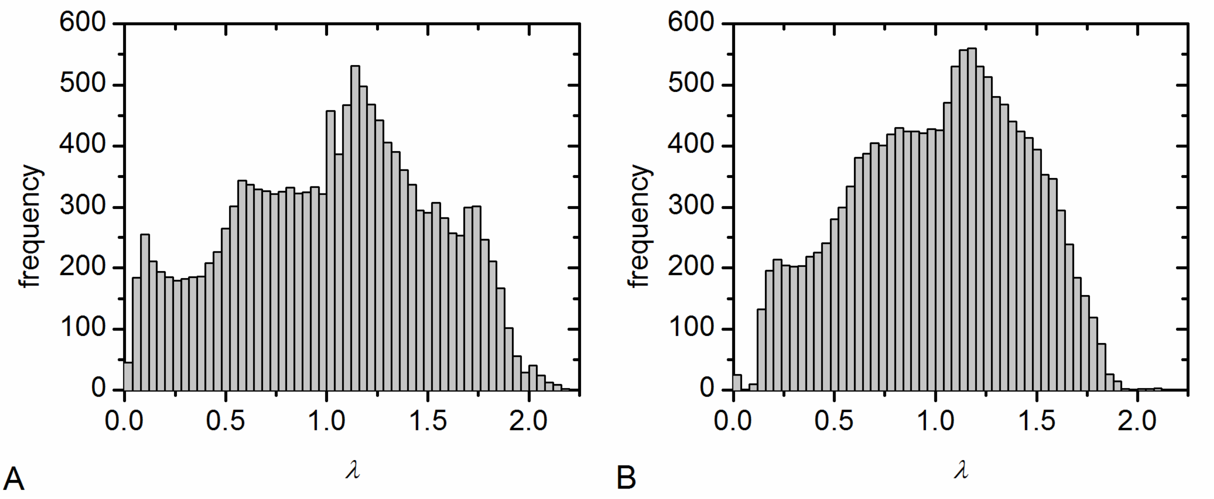

Figure S6. Singular value spectrum of the Laplacian matrix *L* of the network. (A) Summer topology ($\delta = 0.005$) and (B) winter topology ($\delta = 0.01$). Each spectrum was calculated over 25 independent realizations of the network structure.

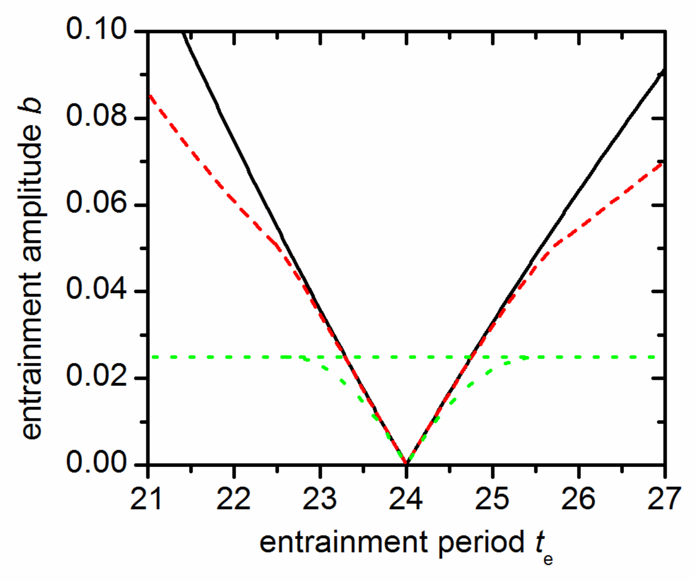

Figure S7. Entrainment borders of the single oscillator under rectangular forcing. (Solid, Black) Rigid oscillator with fast radial relaxation $\lambda >> 1$; (Dashed, Red) Weak, sinusoidal oscillator with slow radial relaxation $\lambda = 0.05$ and $\gamma = 0$; (Dotted, Yellow) Weak, spiking oscillator with slow radial relaxation $\lambda = 0.05$ and $\gamma >> 1$; other parameters were set to $A = 1$ and $t_p = 1/2t_e$. Note that the weak, spiking oscillator is practically entrainable to every period if the entrainment amplitude is above the dotted, yellow horizontal line.

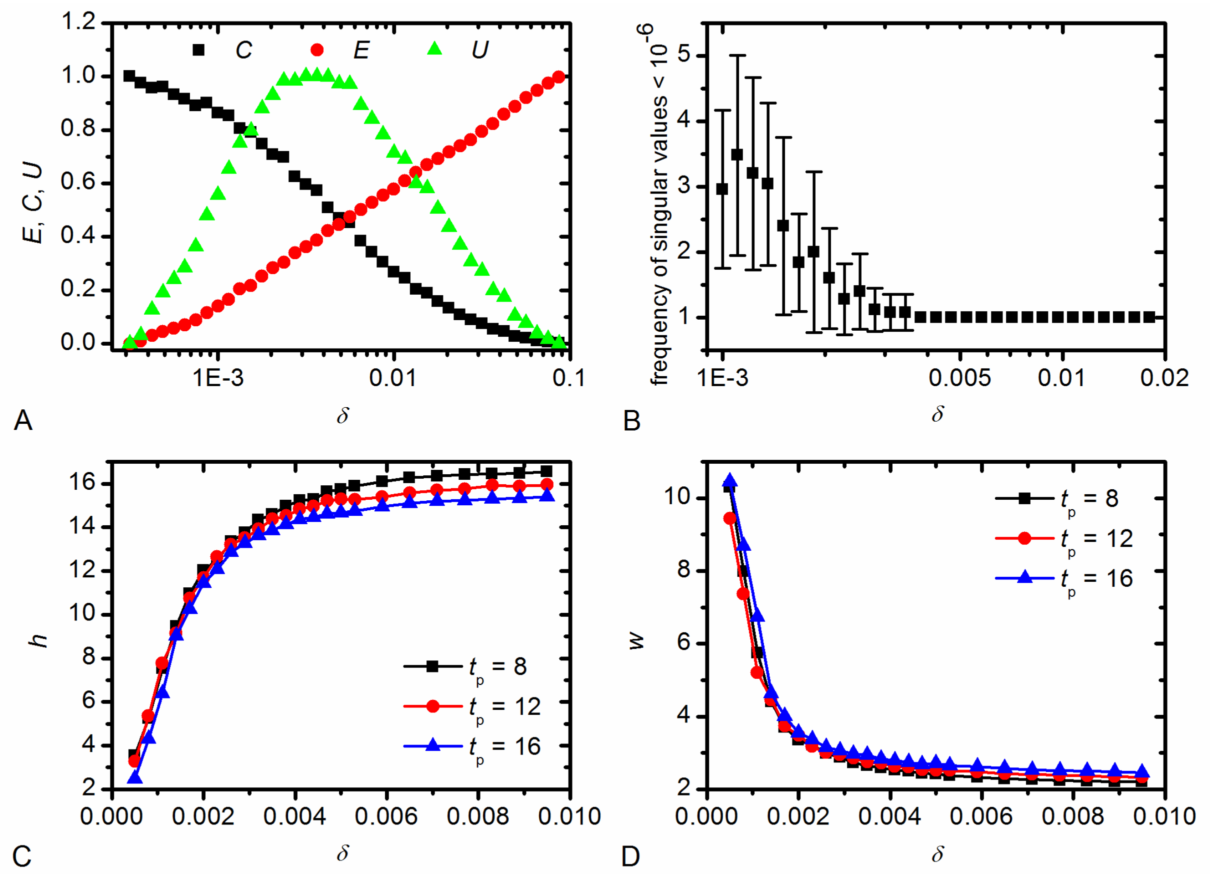

Figure S8. Calculations for the SCN network constituted of N = 1800 neurons. Network properties (A) and spectral analysis (B), the average amplitude h of the mean field signal (C) and its width w (D) as a function of the network parameter δ.

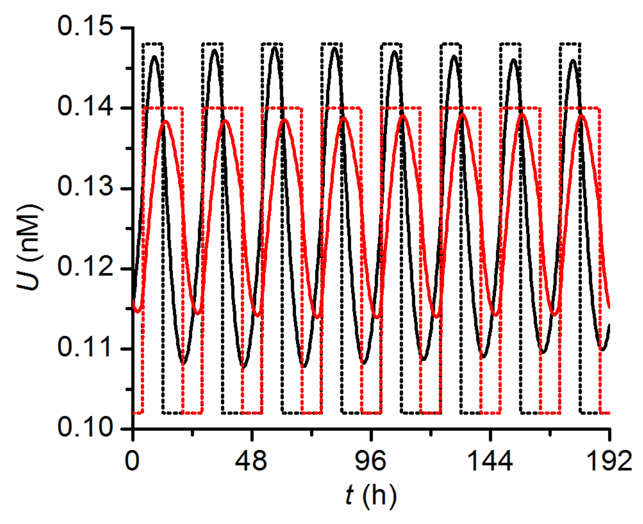

Figure S9.Temporal evolution of the mean-field behavior U of the Goodwin oscillator network. The black and red lines signify the time courses for winter ($\delta = 0.01$, $t_p = 8$) and summer ($\delta = 0.005$, $t_p = 16$) conditions, respectively. The amplitude of the entraining signal is not in scale.

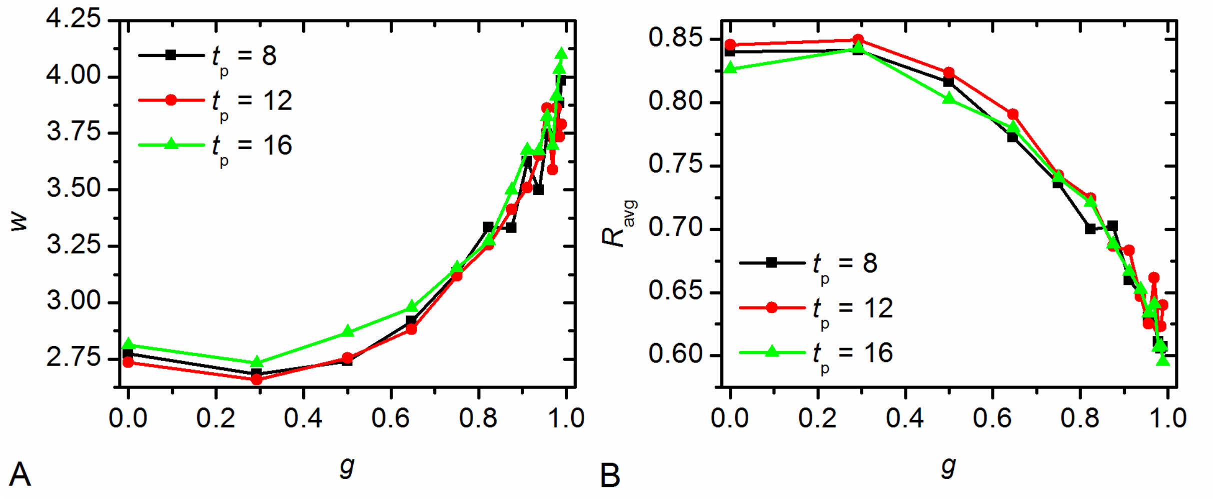

Figure S10. Properties of the electrical activity of the SCN network in the case of a reduced coupling strength of long-range connections. In this simulation, a network of 600 neurons with probability $\delta = 0.01$ for the introduction of long-range conditions is considered. To simulate the transition to summer conditions the coupling strength for half of the long-range connections is reduced by different degrees *g*. The average width of the mean-field signal (A) and the average correlation coefficient (B) are plotted as a function over *g*. In particular, *g*=0 reflects winter conditions and *g*=1 signifies summer conditions, where the coupling constant is set to zero for half of the links reflecting $\delta = 0.005$. Obviously, reducing the coupling strength leads to very similar results as a complete deletion of long-range links.

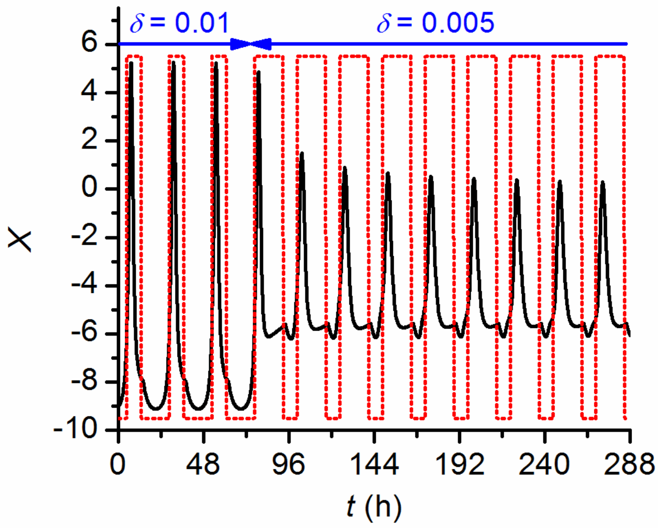

Figure S11. Effects of a rapid transition from winter to summer topology on the electrical activity of the SCN. A network of *N*=600 neurons with winter topology ($\delta = 0.01$) is entrained to short photoperiods. After a certain transient time the network is changed to the summer topology ($\delta = 0.005$) and entrained by long photoperiods.

References

1. Kuramoto Y (1984) Chemical oscillations, waves, and turbulence: Springer-Verlag New York, NY, USA.
2. Rand DA, et al. (2006) Uncovering the design principles of circadian clocks: mathematical analysis of flexibility and evolutionary goals. J Theor Biol 238: 616--635.
3. Bodenstein C, Heiland I, Schuster S (2011) Calculating activation energies for temperature compensation in circadian rhythms. Phys Biol 8: 056007.
4. Ermentrout GB, Kopell N (1991) Multiple pulse interactions and averaging in systems of coupled neural oscillators. J Math Biol 29: 195-217.
5. Bordyugov G, Granada AE, Herzel H (2011) How coupling determines the entrainment of circadian clocks. Eur. Phys. J. B 82: 227-234.
6. McGraw PN, Menzinger M (2007) Analysis of nonlinear synchronization dynamics of oscillator networks by Laplacian spectral methods. Phys Rev E 75: 027104.
7. McGraw PN, Menzinger M (2008) Laplacian spectra as a diagnostic tool for network structure and dynamics. Phys Rev E 77: 031102 .
8. McGraw PN, Menzinger M (2005) Clustering and the synchronization of oscillator networks. Phys Rev E 72: 015101R.
9. Fiedler M (1973) Algebraic Connectivity of Graphs. Czech Math J 23: 298-305.
10. Mohar B (1991) The Laplacian spectrum of graphs. Graph theory, combinatorics, and applications 2: 871-898.
11. Capocci A, et al. (2005) Detecting communities in large networks. Physica A 352: 669-676.
12. Donetti L, Munoz MA (2004) Detecting network communities: a new systematic and efficient algorithm. J Stat Mech: Th Exp **P10012**.
13. Bogoliubov NN (1961) Asymptotic methods in the theory of non-linear oscillations 10: Routledge.
